# Sensitivity of Colorectal Cancer to Arginine Deprivation Therapy is Shaped by Differential Expression of Urea Cycle Enzymes

**DOI:** 10.1038/s41598-018-30591-7

**Published:** 2018-08-14

**Authors:** Constantinos Alexandrou, Saif Sattar Al-Aqbi, Jennifer A. Higgins, William Boyle, Ankur Karmokar, Catherine Andreadi, Jin-Li Luo, David A. Moore, Maria Viskaduraki, Matthew Blades, Graeme I. Murray, Lynne M. Howells, Anne Thomas, Karen Brown, Paul N. Cheng, Alessandro Rufini

**Affiliations:** 1 Department of Genetics and Genome Biology, Leicester Cancer Research Centre, University of Leicester, Leicester, LE2 7LX UK; 2 Department of Pathology and Poultry Diseases, Faculty of Veterinary Medicine, University of Kufa, Kufa, Iraq; 3 Birmingham Women’s Hospital, Birmingham, B15 2TG UK; 4 Department of Pathology, UCL Cancer Centre, UCL, London, UK; 5 Bioinformatics and Biostatistics Support Hub, University of Leicester, Leicester, LE1 7RH UK; 6 Department of Pathology, School of Medicine, Medical Sciences and Nutrition, University of Aberdeen, Foresterhill, Aberdeen AB25, 2ZD UK; 7 Bio-Cancer Treatment International Limited, Hong Kong, Hong Kong

## Abstract

Tumors deficient in the urea cycle enzymes argininosuccinate synthase-1 (ASS1) and ornithine transcarbamylase (OTC) are unable to synthesize arginine and can be targeted using arginine-deprivation therapy. Here, we show that colorectal cancers (CRCs) display negligible expression of OTC and, in subset of cases, ASS1 proteins. CRC cells fail to grow in arginine-free medium and dietary arginine deprivation slows growth of cancer cells implanted into immunocompromised mice. Moreover, we report that clinically-formulated arginine-degrading enzymes are effective anticancer drugs in CRC. Pegylated arginine deiminase (ADI-PEG20), which degrades arginine to citrulline and ammonia, affects growth of ASS1-negative cells, whereas recombinant human arginase-1 (rhArg1peg5000), which degrades arginine into urea and ornithine, is effective against a broad spectrum of OTC-negative CRC cell lines. This reflects the inability of CRC cells to recycle citrulline and ornithine into the urea cycle. Finally, we show that arginase antagonizes chemotherapeutic drugs oxaliplatin and 5-fluorouracil (5-FU), whereas ADI-PEG20 synergizes with oxaliplatin in ASS1-negative cell lines and appears to interact with 5-fluorouracil independently of ASS1 status. Overall, we conclude that CRC is amenable to arginine-deprivation therapy, but we warrant caution when combining arginine deprivation with standard chemotherapy.

## Introduction

Arginine is a semi-essential amino acid in adult mammals that is required for protein synthesis, and is the main substrate for the biosynthesis of nitric oxide, polyamines, proline, creatine, and agmantine^1^. Moreover, arginine, together with leucine, is chiefly responsible for the activation of the mTOR pathway, which in turn stimulates protein translation and other metabolic pathways, such as lipid metabolism and nucleotide biosynthesis^2^. Under physiological conditions, cells satisfy their arginine requirements through direct uptake from the bloodstream and/or through biosynthesis mediated by urea cycle enzymes. Two main enzymes are necessary to produce arginine; ASS1 condensates citrulline and aspartate to form argininosuccinate, which is then converted to arginine and fumarate by argininosuccinate lyase (ASL) (Supplementary Fig. S1)^1^.

A variety of cancers display reduced expression of ASS1 and, to a lesser extent, ASL^3,4^. These tumors are unable to synthesize arginine and are therefore auxotrophic, i.e. depending on external supplementation of arginine for their growth and survival. Resolute efforts to harness this metabolic vulnerability have led to the development of arginine deprivation therapies enabled by the availability of arginine degrading enzymes^5^. Two compounds are currently under clinical evaluation in several malignancies: mycoplasma-derived arginine deiminase (ADI-PEG20) and human arginase-1 (rhArg1peg5000, BCT-100)^6,7^. ADI-PEG20 degrades arginine into citrulline and ammonia, whereas arginase hydrolyses arginine into urea and ornithine. Cells lacking ASS1 or ASL are incapable of recycling citrulline and ornithine and are therefore susceptible to arginine deprivation.

ASS1 expression is transcriptionally regulated. In some tumors, such as glioblastoma, bladder cancer and hepatocellular carcinoma, methylation of the promoter region of the *ASS1* gene mediates its silencing; alternatively, hypoxia-inducible factor 1α (HIF1α)-mediated repression of the *ASS1* promoter has also been reported in cancers such as melanoma^4,8–15^. Traditionally, ASS1 has been adopted as the predictive biomarker for sensitivity to arginine deprivation therapy^5^. Hence, tumors with low expression of ASS1 have been extensively tested for their response to arginine degrading enzymes, whereas tumors with higher expression of ASS1, including CRC^16^, have been deemed ineligible to arginine deprivation therapy. Nonetheless, mounting evidence indicates that modulation of other urea cycle enzymes, such as OTC, can cause arginine auxotrophy and sensitivity to arginine deprivation therapies^4,17–19^.

Here, we unmask a striking arginine auxotrophy in CRC. We show that CRC cell lines are unable to proliferate in arginine-free media and their growth *in vivo* is diminished by administration of an arginine-free diet. Mechanistically, we identify a methylation-independent downregulation of the OTC enzyme in CRC. Reduced OTC expression correlates with sensitivity of CRC cell lines to rhArg1peg5000 treatment *in vitro* and *in vivo*, independently of ASS1 expression. Indeed, resistance to arginase treatment necessitates recycling of ornithine into the urea cycle. This event is mediated by the mitochondrial enzyme OTC, which conjugates ornithine and carmaboylphosphate, a compound synthesized by mitochondrial carmaboylphosphate synthase 1 (CPS1), to form citrulline (Supplementary Fig. S1A). Intriguingly, we also describe a subset of CRC specimens and cell lines expressing low levels of ASS1 and responsive to ADI-PEG20 treatment. Finally, using the Chou-Talalay method for drug combination, we report the feasibility of using arginine deprivation therapy in combination with current chemotherapeutic regimens for CRC. Overall, our results reveal that CRC is amenable to treatment with arginine-deprivation therapy.

## Results

### CRC cell lines display arginine auxotrophy

**Figure 1:**
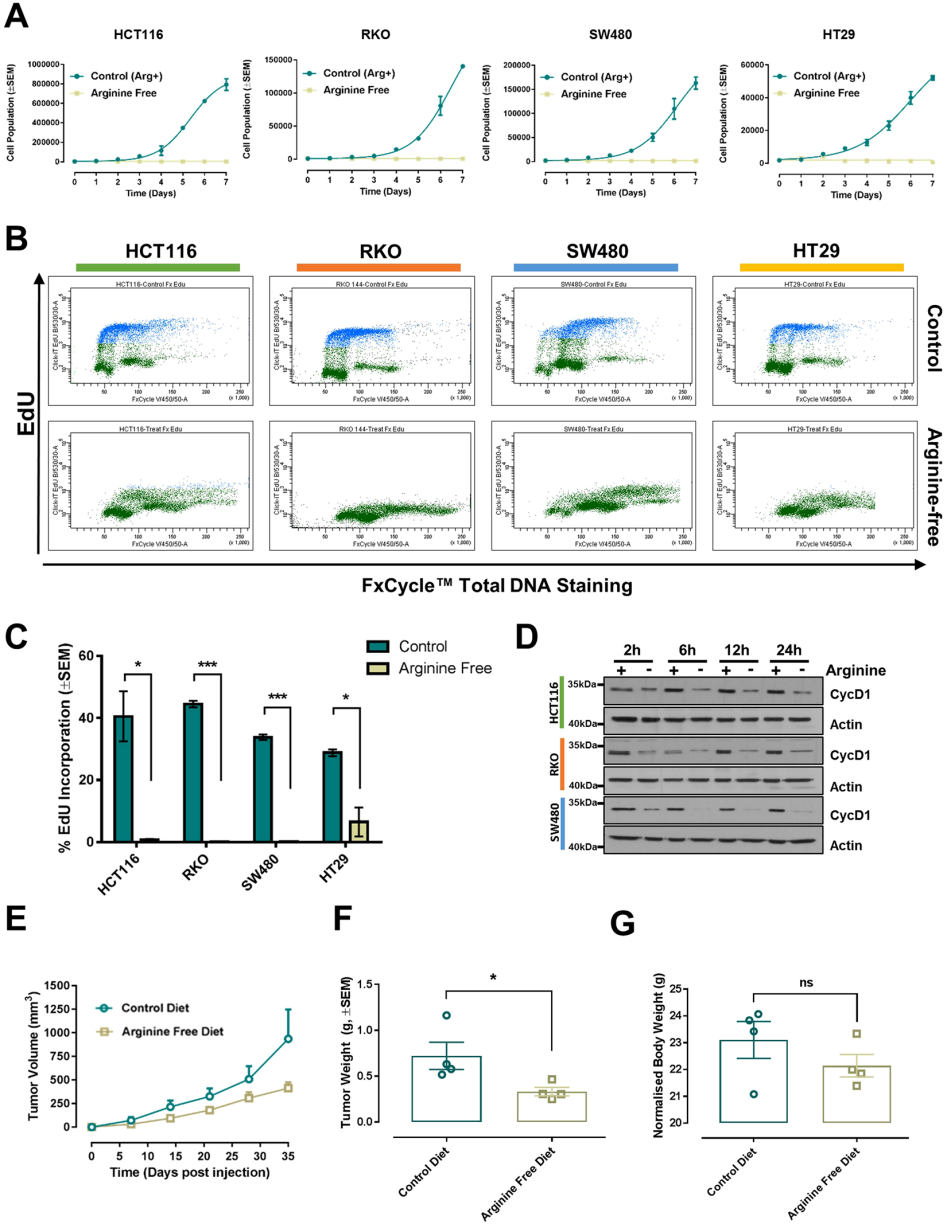
Arginine auxotrophy in CRC. (**A**) Growth curves of the indicated cell lines with or without arginine supplementation. Data are presented as mean ± SEM of three independent experiments. (**B**) Representative flow cytometry scatterplots of EdU incorporation in HCT116, RKO, HT29 and SW480 CRC cell lines grown in control medium or arginine-free medium for 24 h. EdU was measured using the Click-iT® EdU Alexa Fluor® kit and total DNA stained using FxCycle™ Violet Stain. (**C**) Quantification of EdU incorporation from three independent experiments. Data are presented as mean ± SEM, two-tailed t-test. *P < 0.05, ***P < 0.001. (**D**) Western blot analysis of Cyclin D1 (CycD1) protein expression in HCT116, RKO and SW480 CRC cell lines following arginine deprivation for the indicated times. Actin was used as endogenous loading control. Original western blots are reported in Supplementary Fig. S15. (**E)** Graph showing the growth of xenografted HCT116 cells in immunocompromised mice fed a control diet or an arginine-free diet. Data are plotted as mean ± SEM and were analyzed using mixed linear regression analysis (P = 0.03; P < 0.05 indicates a statistically significant difference in tumor growth rate between control and treated animals over time). (**F)** Weight of excised tumor measured at endpoint. Data are plotted as mean ± SEM. *P < 0.05, two-tailed t-test (n = 4 animal per group). (**G)** Animal body weights at endpoint plotted as mean ± SEM. No statistical differences were detected between the two diet groups, two-tailed t-test (n = 4 animals per group).

To assess whether external arginine supplementation is necessary to sustain CRC growth, we cultured CRC cell lines in arginine-free medium or in a control medium containing all amino acids. Notably, all cell lines tested failed to grow in the absence of arginine (Fig. 1A), indicating auxotrophy. The halted growth was accompanied by an arrest of DNA replication (Fig. 1B and C) and decreased expression of the cell cycle marker Cyclin D1 (Fig. 1D), and was reverted upon arginine repletion (Supplementary Fig. S1B). Consistent with the role of arginine in regulating mTOR signaling, we observed decreased mTOR activity, as indicated by the loss of the higher molecular weights rapamycin-sensitive phosphosites of the 4E-BP1 protein^20^ (Supplementary Fig. S2).

To investigate whether the identified arginine addiction prevails *in vivo*, HCT116 cells where subcutaneously injected in immunocompromised athymic nude mice. After injection, mice were randomized to an arginine-free diet or a control diet (0.83% arginine) and tumor growth measured by calipers. Nutritional deprivation of arginine was effective in slowing tumor growth, as demonstrated by diminished tumor volume and weight (Fig. 1E and F and Supplementary Fig. S3). Notably, dietary arginine deprivation did not affect animal body weight during the 35-day duration of the experiment (Fig. 1G). Overall, these findings identify arginine auxotrophy as a metabolic vulnerability in CRC.

### Downregulation of OTC and ASS1 expression in CRC

**Figure 2:**
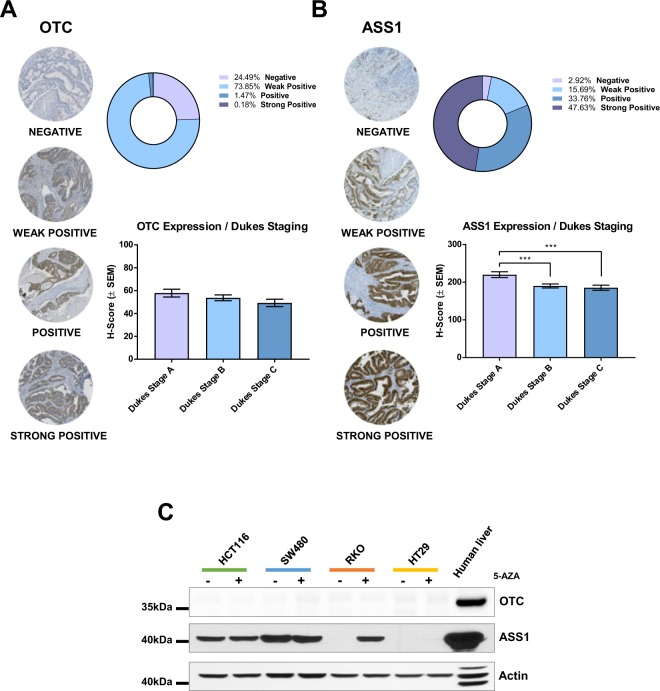
Reduced expression of OTC and ASS1 in CRC. (**A**) OTC and (**B**) ASS1 H-score were assessed on CRC TMA. Representative histo-spots are shown on the left. The bar graphs indicate H-score distribution according to Duke’s stage, whereas the distribution of protein expression within the whole TMA cohort is reported in the pie chart. *P < 0.05 (**C)** Western blot analysis of OTC and ASS1 after 72 h treatment with 5 µM 5-Azacytidine (5-AZA). Human liver extract was used as positive control for urea cycle enzymes, actin was used as endogenous loading control. Original western blots are reported in Supplementary Fig. S15.

To investigate the mechanism responsible for the arginine dependence of CRC, we scored a TMA cohort, consisting of over 600 cases of CRC (Supplementary Table S1), for expression of OTC and ASS1 proteins. OTC expression was low or absent in virtually all cases. Interestingly, we also identified a subset of CRC patients (~18%) that expressed low or undetectable ASS1 levels (Fig. 2A and B, Supplementary Fig. S4).

Low expression of OTC and ASS1 was then confirmed by assessment of protein levels in a panel of CRC cells lines (Fig. 2C). No cell line showed evidence of OTC expression, which was promptly detected in control human liver. With regard to ASS1, its expression pattern in cell lines mirrored the heterogeneity observed in TMA; SW480 cells showed sustained expression of the enzyme, HCT116 cells expressed ASS1 moderately, whereas the expression of the enzyme was absent in HT29 and RKO cell lines. Finally, we analyzed gene expression datasets using the online platforms CancerMA and Oncomine^21,22^ and we confirmed downregulation of the *OTC* gene expression in colorectal tumors (Supplementary Fig. S5).

CpG island methylator phenotype (CIMP), which causes epigenetic gene silencing through methylation of cytosine residues at CpG-rich DNA sequences, contributes to the progression of CRC^23^. Both RKO and HT29 cells are CIMP positive^24^. Hence, in the light of the reported role of promoter methylation in mediating silencing of the genes coding for the urea cycle enzymes, we investigated whether reversion of DNA methylation with the DNA methylase inhibitor 5-azacytidine (5-AZA) could rescue expression of ASS1 and OTC. Our results indicate that ASS1 suppression in RKO is indeed methylation-dependent, whereas CIMP is unlikely to mediate the loss of ASS1 in HT29. On the other hand, 5-AZA treatment did not rescue OTC expression in any of the cell line analyzed, suggesting that the regulation of *OTC* gene is not regulated through promoter methylation (Fig. 2C).

### CRC cell lines are sensitive to arginine deprivation by rhArg1peg5000

**Figure 3:**
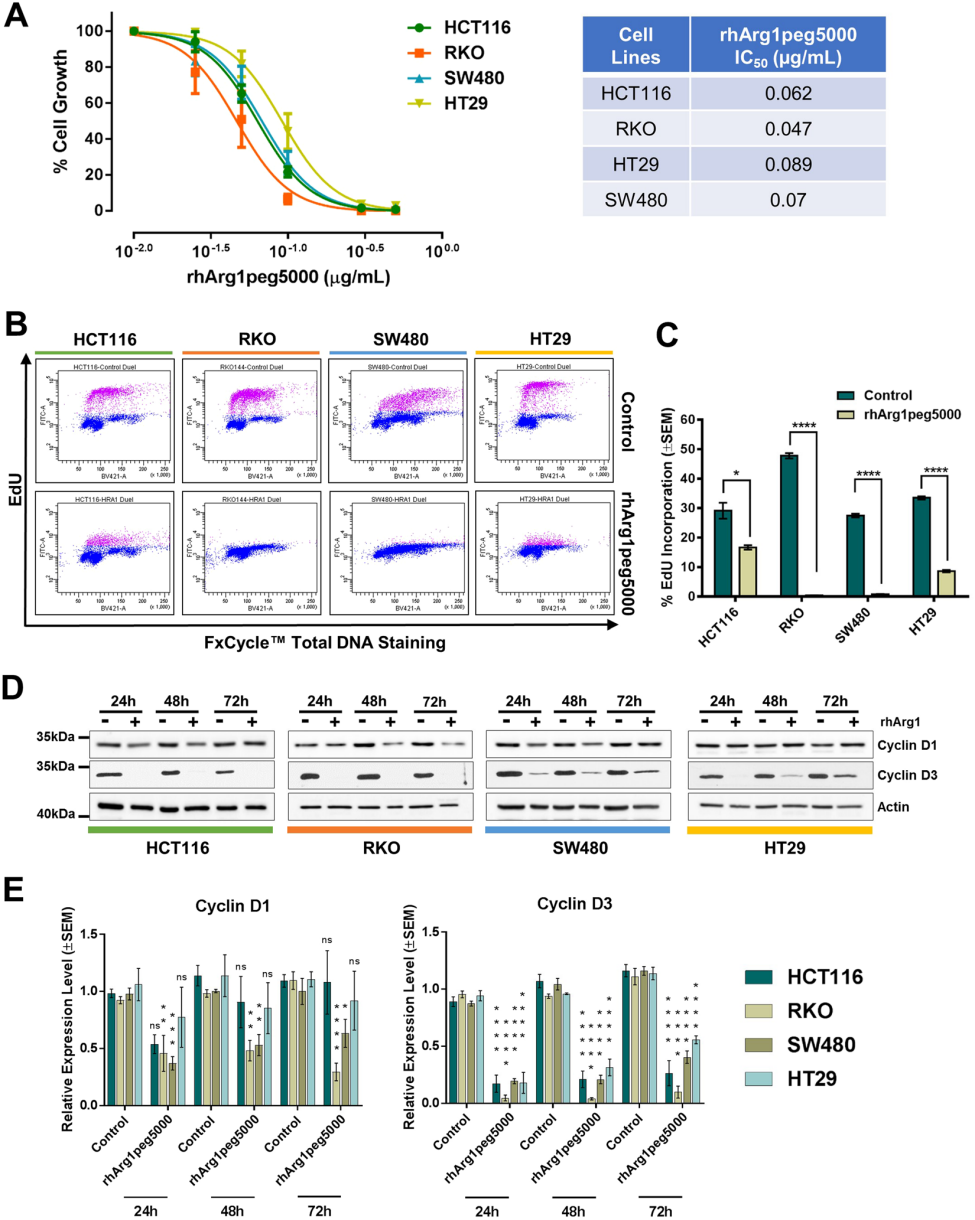
CRC cells are sensitive to arginase treatment. (**A**) Dose-Response Non-Linear Regression Curves and IC_50_ values of the indicated CRC cell lines treated with the rhArg1peg5000. The percentage (%) of cell growth was calculated relative to the cell numbers in corresponding PBS-treated control samples, which was selected as 100%. IC_50_ values were obtained from non-linear regression analysis of concentration of the drug vs response curves. The results were obtained from three independent experiments. Quadruplicate samples were assessed for cell growth after a 6-day period of treatment by cell counting for each individual experiment. The error bars represent ± SEM. (**B)** Representative flow cytometry scatterplots of EdU incorporation in HCT116, RKO, HT29 and SW480 CRC cell lines after 72 h treatment with rhArg1peg5000 (0.5 μg/mL) or PBS-vehicle control. EdU was measured using the Click-iT® EdU Alexa Fluor® kit and total DNA stained using FxCycle™ Violet Stain. (**C)** Quantification of EdU incorporation from three independent experiments. Data are presented as mean ± SEM. *P < 0.05, ****P < 0.0001, two-tailed t-test. **(D)** Western blot analysis of the cell cycle markers Cyclin D1 and D3 in cells treated for the indicated time with rhArg1peg5000 (0.5 μg/mL). Actin was used as endogenous loading control. (**E**) Quantification of Cyclin D1 and D3 protein expression from triplicate experiments. Original western blots are reported in Supplementary Fig. S15. Data are presented as mean ± SEM. *P < 0.05, **P < 0.01, ***P < 0.001, ****P < 0.0001, two-way ANOVA.

Prompted by the marked arginine addiction of CRC cell lines and the impaired expression of urea cycle enzymes in cancer patients, we tested the feasibility of targeting CRC using pharmacological arginine deprivation. Because of the prevalent loss of OTC expression, we investigated whether depletion of arginine using rhArg1peg5000 could affect CRC cell growth. We found that all cell lines tested, independently of ASS1 expression, showed a robust, dose-dependent decrease in cell number upon rhArg1peg5000 treatment, with IC50 concentrations lower than 0.1 μg/ml (Fig. 3A). To explore the mechanism responsible for the observed growth reduction, we measured cellular proliferation using the EdU incorporation assay and found that arginine depletion led to a sharp decrease in DNA synthesis (Fig. 3B and C), indicative of proliferative arrest. In agreement with this result, arginine-deprived cells displayed reduced expression of the cell cycle markers Cyclin D3 and Cyclin D1 (Fig. 3D and E). Notably, rhArg1peg5000 treatment elicited expression of ASS1 in HCT116, RKO and HT29 (Supplementary Fig. S6). ASS1 expression also correlated with enhanced c-Myc expression in RKO and HT29 cells, in line with data showing c-Myc-mediated ASS1 re-expression in cells treated with arginine deprivation therapy^25^. Intriguingly, no compensatory expression of OTC was observed during the 72 h treatment (Supplementary Fig. S7).

Few floating cells were observed in the plates treated with rhArg1peg5000, possibly signifying a marginal induction of cell death. Indeed, the apoptotic marker cleaved-PARP was induced in arginine-deprived HCT116 and RKO cells (Supplementary Fig. S8).

Next, we investigated whether rhArg1peg5000 treatment affects the mTOR pathway in a way similar to that observed in arginine-free medium. To this end, the phosphorylation status of two main mTOR downstream targets, the ribosomal S6 protein and 4E-BP1, was investigated by western blotting over a short (up to 8 h) and long (up to 72 h) time course. However, we did not observe durable and consistent changes in phosphorylation levels of mTOR targets (Supplementary Fig. S9).

### rhArg1peg5000 reduces tumor growth *in vivo*

**Figure 4:**
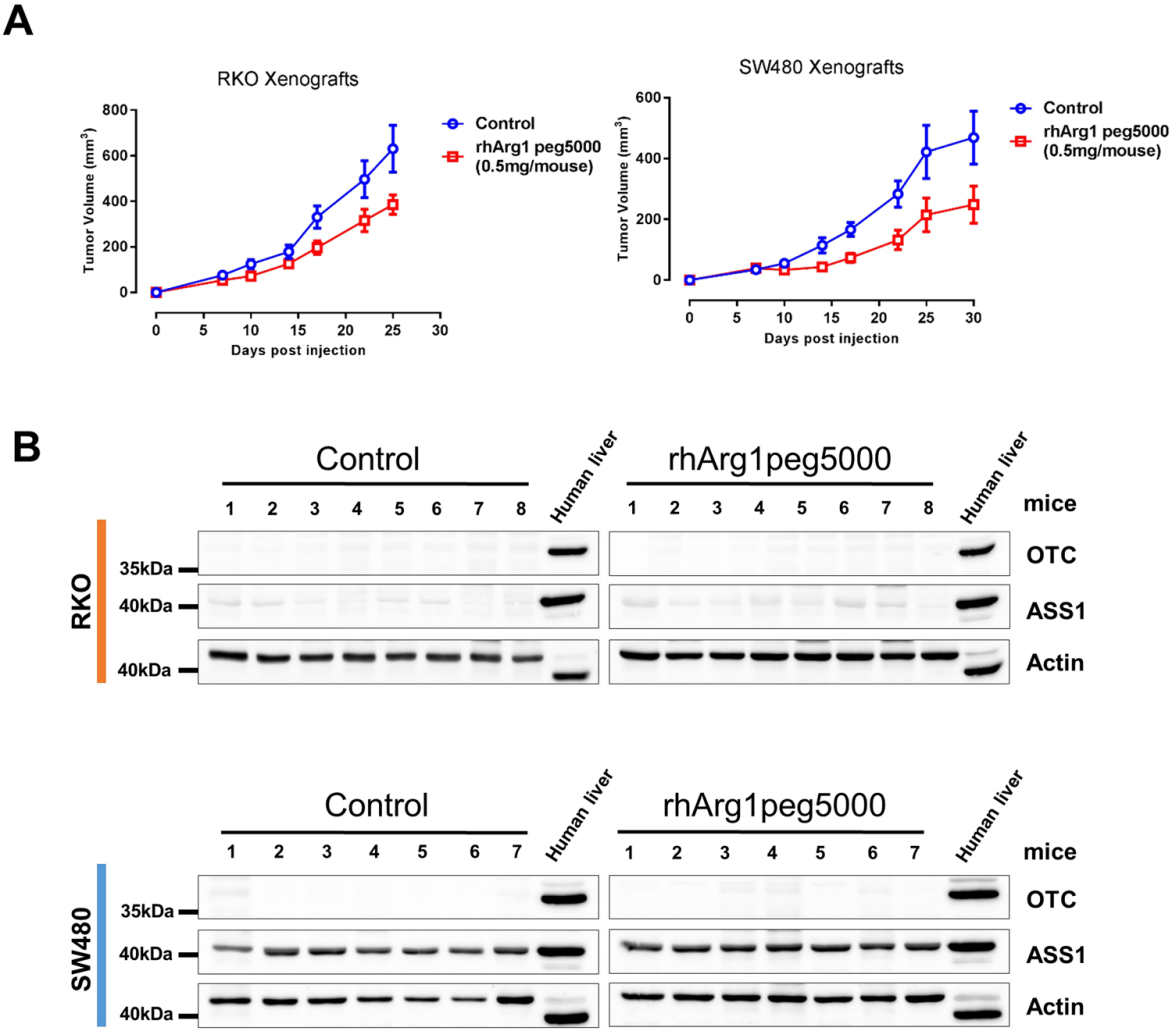
Pharmacological depletion of arginine using rhArg1peg5000 reduces tumor growth *in vivo*. (**A**) Tumor growth in athymic nude mice subcutaneously injected with 10^6^ RKO or SW480 CRC cells. Mice were randomized into Control (n = 8) and Treatment (n = 8) groups. Treated mice were administered intraperitoneally with 0.5 mg of rhArg1peg5000/animal twice a week, while control mice were injected with an equal volume of PBS. Data are plotted as mean ± SEM and were analyzed using mixed linear regression analysis (P = 0.012 for RKO and P = 0.03 for SW480, P < 0.05 indicates a statistically significant difference in tumor growth rate between control and treated animals over time). (**B**) Western blotting of lysates from tumor xenografts for assessment of urea cycle enzymes OTC and ASS1. Human liver lysate was used as a positive control and actin as endogenous loading control. Original western blots are reported in Supplementary Fig. S15.

To test whether arginine deprivation mediated by rhArg1peg5000 could impair tumor growth *in vivo*, we subcutaneously implanted ASS1-negative RKO cells and ASS1-positive SW480 cells into the hind flanks of immunocompromised athymic nude mice, which were then randomized to twice a week treatment schedule (0.5 mg of rhArg1peg5000 per animal) or vehicle control. Arginase administration significantly slowed tumor growth in animals into which either cell line had been implanted (Fig. 4A). Pegylated arginine was well tolerated and there was no difference in body weight recorded between control and treated animals (Supplementary Fig. S10).

Western blotting analysis with antibodies selective for the human protein confirmed robust expression of ASS1 in SW480-derived tumors, as well as ASS1 negativity in RKO xenograft samples (Fig. 4B). Notably, no expression of OTC was detected in tumor isolated from rhArg1peg5000 treated animals (Fig. 4B). These findings suggest that tumors did not acquire resistance against arginase through re-expression of urea cycle enzymes, at least in the time frame of these experiments.

Overall, these data indicate that CRC cells are sensitive to pharmacological depletion of arginine via pegylated-arginase in an ASS1-independent fashion.

### CRC cell lines are sensitive to arginine deprivation by ADI-PEG20

**Figure 5:**
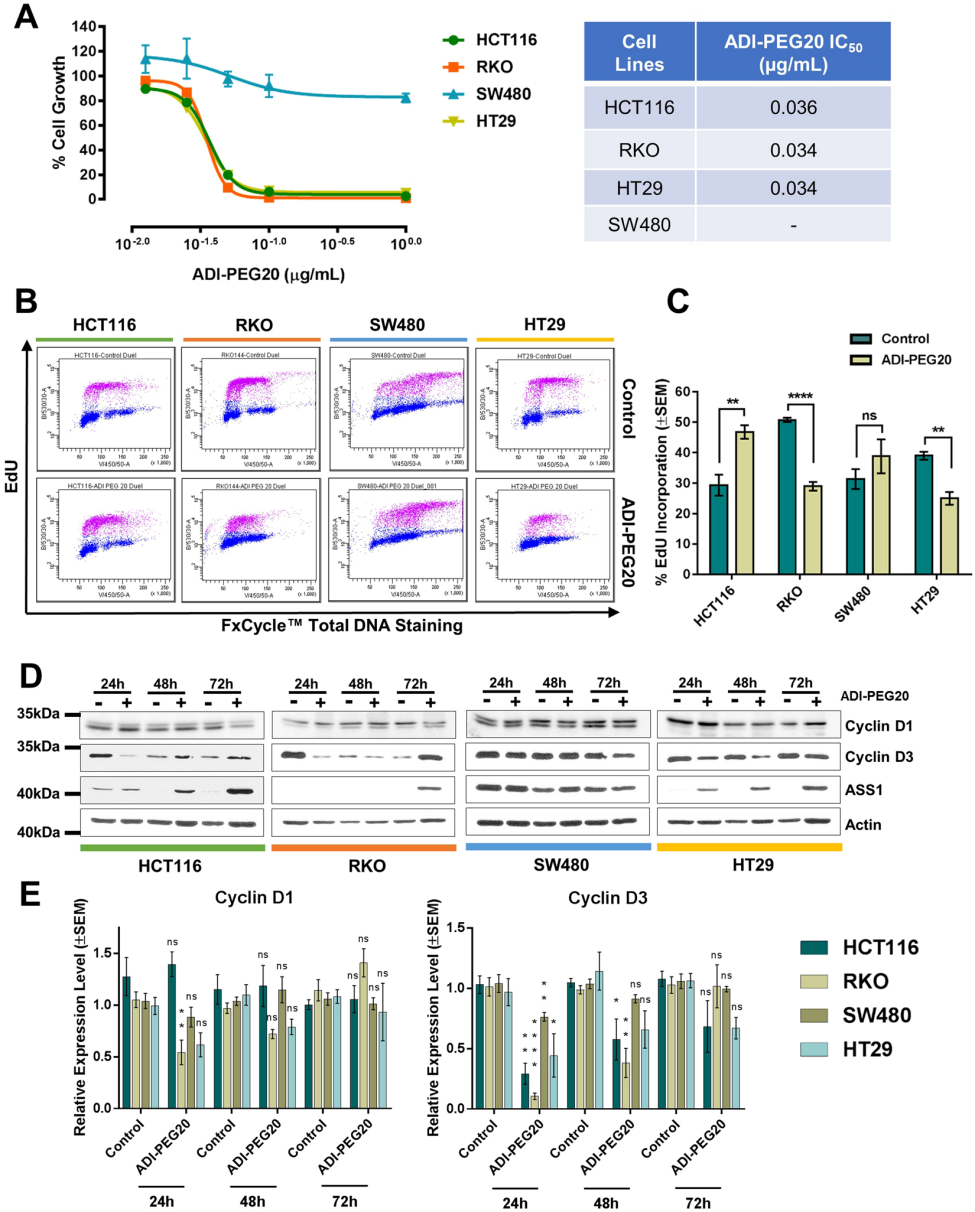
CRC cells are sensitive to arginine deiminase treatment. (**A**) Dose-Response Non-Linear Regression Curves and IC_50_ values of the indicated CRC cell lines treated with the ADI-PEG20. The percentage (%) of cell growth was calculated relative to the cell numbers in corresponding PBS-treated control samples, which was selected as 100%. IC_50_ values were obtained from non-linear regression analysis of concentration of the drug vs response curves. The results were obtained from three independent experiments. Quadruplicate samples were assessed for cell growth after a 6-day period of treatment by cell counting for each individual experiment. The error bars represent ±SEM. (**B)** Representative flow cytometry scatterplots of EdU incorporation in HCT116, RKO, HT29 and SW480 CRC cell lines after 72 h treatment with ADI-PEG20 (1 μg/mL) or PBS-vehicle control. EdU was measured using the using Click-iT® EdU Alexa Fluor® kit and total DNA stained using FxCycle™ Violet Stain. (**C**) Quantification of EdU incorporation from three independent experiments. Data are presented as mean ± SEM. *P < 0.05, ****P < 0.0001, two-tailed t-test. (**D**) Western blot analysis of the cell cycle markers Cyclin D1 and D3 and the urea cycle enzyme ASS1 in cells treated for the indicated time with ADI-PEG20 (1 μg/mL). Actin was used as endogenous loading control. Original western blots are reported in Supplementary Fig. S15. (**E**) Quantification of Cyclin D1 and D3 protein expression from triplicate experiments. Data are presented as mean ± SEM. *P < 0.05, **P < 0.01, ***P < 0.001, ****P < 0.0001, two-way ANOVA.

As described above, RKO and HT29 cell lines do not express detectable ASS1 protein, and around 20% of CRC patients present with no or low expression of this biomarker (Fig. 2B and C). Hence, we investigate whether the mycoplasma-derived enzyme arginine deiminase could represent an alternative treatment opportunity for those patients. As expected, ASS1-positive SW480 cell lines were resistant to treatment with ADI-PEG20 *in vitro*, whereas ASS1-negative HT29 and RKO showed an exquisite sensitivity to ADI-PEG20-mediated arginine deprivation, with an identical IC50 of 0.034 μg/mL (Fig. 5A). In agreement with recently reported findings^26,27^, HCT116 growth was greatly reduced by ADI-PEG20, despite detectable levels of ASS1 (Fig. 5A). The reason for the HCT116 sensitivity to ADI-PEG20 is unclear, as HCT116 cells also express ASL enzyme^26^. Others have attributed the effect of ADI-PEG20 treatment to downregulation of the mTOR pathway and induction of the unfolded protein response^26^, a possibility corroborated by the reduced phosphorylation of the mTOR targets 4E-BP1 and S6 after 72 h of arginine deiminase treatment (Supplementary Fig. S11).

When proliferation was assessed after 72 h exposure to ADI-PEG20, we noticed a significant reduction of EdU incorporation only in ASS1-negative cells, but not in ASS1-positive SW480 and HCT116 (Fig. 5B and C). Further analysis indicated that, in all ADI-PEG20 sensitive cell lines, expression of the cell cycle markers Cyclin D1 and D3 was reduced 24 h to 48 h after drug treatment, but had returned to normal levels at the time point of the cell cycle analysis (72 h) (Fig. 5D and E). Similarly, the expression of ASS1 was boosted by ADI-PEG20 treatment (Fig. 5D). These findings could explain why DNA synthesis was still substantial at the 72 h time point of arginine deprivation, and they indicate the possibility of rapidly ensuing resistance.

### ADI-PEG20 reduces tumor growth *in vivo*

**Figure 6:**
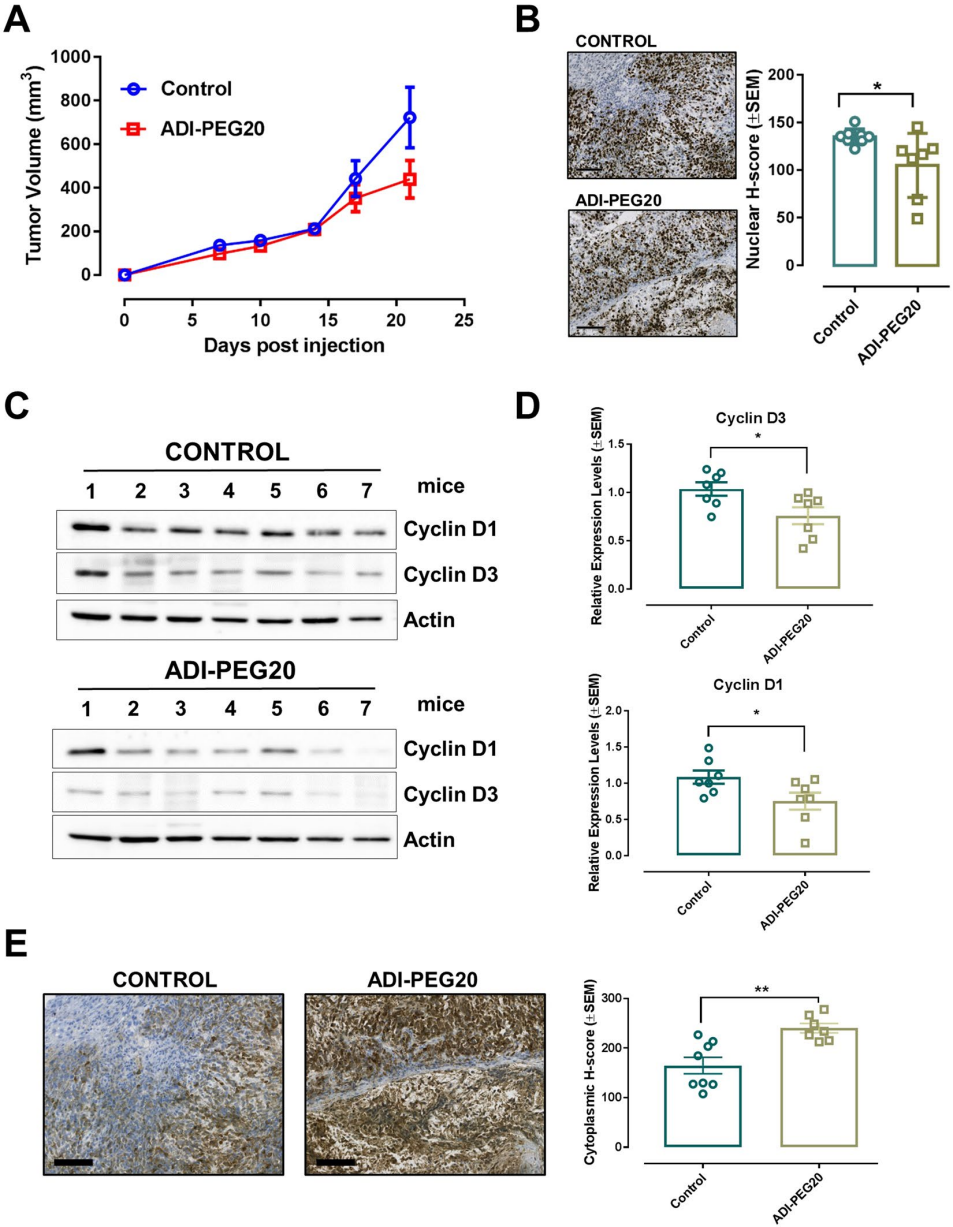
Pharmacological depletion of arginine using ADI-PEG20 reduces tumor growth *in vivo*. (**A**) Tumor xenografts growth in athymic nude mice subcutaneously injected with 10^6^ RKO CRC cells. Mice were randomized into Control (n = 8) and Treatment (n = 8) groups. Treated mice were administered intraperitoneally (IP) with 5IU of ADI-PEG20 per animal per week, while control mice were injected with equal volume of PBS. Data are plotted as mean ± SEM and were analyzed using mixed linear regression analysis (P = 0.045, P < 0.05 indicates a statistically significant difference in tumor growth rate between control and treated animals over time). (**B)** Representative images of ki67-stained xenograft tumors and ki67 proliferation index of tumors from vehicle-treated and ADI-PEG20-injected animals. Data are presented as mean ± SEM (n = 7 animals per group). *P < 0.05, two-tailed t-test. Size bars = 100 μm. (**C)** Western blot analysis of the cell cycle markers Cyclin D1 and D3 and urea cycle enzyme ASS1 in xenograft tumors isolated from PBS-injected controls and ADI-PEG20-treated animals. Actin was used as endogenous loading control. Original western blots are reported in Supplementary Fig. S15. (**D)** Graph bars show quantification of Cyclins levels using Image J software. Data are presented as mean ± SEM (n = 7 animals per group). *P < 0.05, two-tailed t-test. (**E)** Representative images of ASS1-stained xenograft tumors and ASS1 H-score index of tumors from vehicle-treated and ADI-PEG20-injected animals. Data are presented as mean ± SEM (n = 7 animals per group). **P < 0.01, two-tailed t-test. Size bars = 100 μm.

To determine whether ADI-PEG20 could slow tumor growth *in vivo*, ASS1-negative RKO cells were subcutaneously implanted into nude mice, which were then randomly allocated to either treatment (5IU of ADI-PEG20/animal/week) or vehicle control groups. ADI-PEG20 administration reduced tumor volume (Fig. 6A), although treated mice failed to gain body weight (Supplementary Fig. S12). The reduced tumor volume, was accompanied by a moderate but significant decrease in the Ki67 proliferation index (Fig. 6B), as well as Cyclin D1 and D3 expression in ADI-PEG20 treated xenografts and (Fig. 6C and D). Finally, as observed *in vitro*, ASS1 protein levels were increased in tissue isolated from arginine-deprived animals (Fig. 6E).

Overall, these data indicate that ADI-PEG20 treatment reduces *in vivo* tumorigenicity of ASS1-deficient CRC cells.

### Combination of arginine deprivation with traditional chemotherapy

Chemotherapy based on the platinum compound Oxaliplatin and the antimetabolite 5-FU remains a key combination for first line treatment of metastatic CRC or in adjuvant settings^28,29^. Several publications suggest that ADI-PEG20 has a synergistic effect when combined with platinum-based compounds^9,15,30–32^. Hence, we endeavored to assess whether combining arginine deprivation therapy with chemotherapy might improve the treatment of CRC. To this end, we investigated effects of combinations on cell growth employing the Chou-Talalay method and the CompuSyn software^33^ (Table 1, Supplementary Figs S13 and S14). Arginase treatment mostly resulted in antagonism (Combinatorial Index, CI > 1) when tested with either Oxaliplatin or 5-FU. Additive effects (CI = 1) were observed under a minority of conditions. Conversely, ADI-PEG20 in combination with Oxaliplatin elicited synergistic growth inhibition in the ASS1-negative cell lines RKO and HT29 (CI < 1). Of note, with the exception of ADI-PEG20-treated HT29, we never observed synergism between 5-FU and arginine deprivation therapy, rather, antagonism was the most common outcome of co-treatments.

**Table 1:** Combination studies between ADI-PEG20 or rhArg1peg5000 and chemotherapy.

	ADI-PEG20		rhArg1peg5000	
	IC50	IC90	IC50	IC90
**5-FU**				
HCT116	1.42	1.25	1.73	1.63
SW480	**—**	**—**	1.76	1.08
RKO	1.18	1.1	1.05	1.09
HT29	0.89	**0.80**	1.41	1.25
**Oxaliplatin**				
HCT116	1.24	1.46	1.92	1.60
SW480	—	—	1.54	1.22
RKO	**0.61**	**0.80**	1.89	1.59
HT29	**0.61**	**0.80**	1.33	1.09

CI values are reported for both IC_50_ and IC_90_ combinations in the indicated cell lines. CI < 1 indicates synergism, CI = 1 indicates addictive effect, CI > 1 indicates antagonism. Cells were counted after 6 days of treatment and data obtained from diagonal constant ratio combinations were analyzed for synergism, additivity or antagonism using the CompuSyn Software (CompuSyn Inc.) according to the Chou and Talalay method.  Bold highlights significant synergisms (P < 0.05).

Overall, these combinatorial studies outline a complex interaction between arginine deprivation and chemotherapy. Whereas arginase treatment often appears to be antagonistic with chemotherapeutic drugs, ADI-PEG20, in some circumstances, displays synergism in ASS1-deficient cells.

## Discussion

ADI-PEG20 and rhArg1peg5000 (BCT-100) are under intense clinical testing in numerous malignancies. ADI-PEG20 degrades arginine into citrulline and ammonia, whereas rhArg1peg5000 generates ornithine and urea. Resistance of cells against these drugs depends on the expression of the urea cycle enzymes which are capable of resynthesizing arginine from the catabolic products. Expression of ASS1 and ASL is necessary for resistance to ADI-PEG20 monotherapy^34^, whereas resistance to arginase requires the additional expression of the enzyme OTC^19^. When investigating biomarkers of arginine auxotrophy in CRC, we recorded a consistently poor expression of the urea cycle enzyme OTC in CRC specimens. In mammals OTC protein is expressed only in the liver and intestine^35^ and catalyzes the reaction between carbamoyl-phosphate and ornithine to generate citrulline^1^. This reaction is enabled by the Carbamoyl-Phosphate Synthase 1 (CPS1)-mediated biosynthesis of carbamoyl-phosphate, which channels nitrogen from glutamine into the urea cycle. The reason for OTC downregulation in CRC remains unclear. Loss of ASS1 expression has been associated with poor prognosis in bladder cancer and glioblastoma^4,8^ and recent data indicate that ASS1 downregulation supports cancer growth allowing aspartate channeling into pyrimidine biosynthesis^36^. With regard to regulation of urea cycle enzymes, CRC is anomalous as OTC downregulation is often accompanied by high expression of ASS1^16^. In CRC, ASS1 has been proposed to support proliferation of cancer cells, possibly through the enhancement of the glycolytic flux^37^. Interestingly, preliminary evidence suggests that CPS1 is also downregulated in advanced CRC^38^, intimating that the entire pathway leading to citrulline production is compromised. The reason for this phenomenon is not clear, but it is tempting to speculate that lack of OTC and CPS1 expression enable recycling of nitrogen for reactions such as nucleotide, amino acid, or polyamine biosynthesis. Whatever the benefit, OTC downregulation renders cancer cells sensitive to treatment with human arginase, independently of ASS1 expression. Indeed, our *in vivo* studies show that pegylated arginase diminish the growth of both ASS1-positive and negative CRC cells. This finding is particularly interesting, as the potential therapeutic benefit of arginase in the treatment of solid tumors has hitherto been rarely investigated^19,39,40^, even though it has been well-established in blood malignancies^17,41–44^.

Intriguingly, our TMA analysis indicates that in a fraction of CRC patients ASS1 is poorly expressed or undetectable, an occurrence mirrored by RKO and HT29 cell lines, which both demonstrated significant sensitivity to arginine deprivation via ADI-PEG20 treatment *in vitro* and *in vivo*. These data intimate that a subset of CRC patients may be eligible to treatment with arginine deiminase, although the rapid reappearance of ASS1 in cells treated with ADI-PEG20 monotherapy is suggestive of a rapid adaptation and fast ensuing resistance. Indeed, restored expression of ASS1 has been reported in patients treated with single agent ADI-PEG20^45^ and it is a common mechanism of acquired resistance against arginine deprivation^25,34,46^. Our data indicate that a similar re-expression of ASS1 activity is likely to occur in CRC, but, surprisingly, we did not observe re-expression of OTC, at least under the experimental conditions used. Currently, the mechanism mediating this persistent loss of OTC expression remains elusive, although experiments with the DNA methylase inhibitor suggest that it is likely to be independent of promoter methylation.

In several malignancies, ADI-PEG20 synergizes with platinum based chemotherapy^9,15,30,31^, and a recent clinical trial reinforces the relevance of this interaction in combination with the folate antagonist pemetrexed in thoracic malignancies^32^. Chemotherapy of CRC relies on oxaliplatin and 5-FU (FOLFOX), and an ongoing clinical trial (NCT02102022) aims at treating patients with advanced gastrointestinal malignancies, including CRC, with a combinations of ADI-PEG20 and FOLFOX therapy. This projected trial inspired us to search for synergistic interactions between arginine degrading enzymes and the chemotherapeutic drugs oxaliplatin and 5-FU. Unexpectedly, arginase and arginine deiminase behaved dissimilarly in the combination studies. We failed to identify synergistic effects of rhArg1peg5000, which mostly antagonized both oxaliplatin and 5-FU, whereas ADI-PEG20 showed either synergistic or antagonistic effects, depending on drug used in the combination and cell line. We observed synergism between ADI-PEG20 and oxaliplatin in ASS1-deficient CRC cell lines, but we only observed synergism with 5-FU in ASS-1-negative HT29 cells. Therefore, our data warrant caution when using arginine deprivation therapy in combination with traditional chemotherapy in CRC, as patients’ responses might differ substantially, perhaps consistent with the well-established molecular heterogeneity of colon malignancies^47^. On the other hand, the evidence of synergistic interactions intimates interesting therapeutic opportunities, at least for a subset of patients. Indeed, in a clinical study combining ADI-PEG20 with nab-paclitaxel and gemcitabine in pancreatic cancer, objective results were observed in both ASS1-proficient and -deficient patients^48^. This suggests that ASS1-deficiency may be less relevant if ADI-PEG20 is combined with other anticancer agents. Also, some malignant cell lines downregulate ASS1 expression when exposed to ADI-PEG20^34^, suggesting that they may be more sensitive to ADI-PEG20 monotherapy, as well as ADI-PEG20 combination therapy. Furthermore, ADI-PEG20 inhibits migration of endothelial cells, even when co-cultured with ASS1 proficient tumors, at least in part by altering the composition and distribution of filamentous actin and attenuating tumor-produced vascular endothelial growth factor^49^. This demonstrates that, in some cases, ADI-PEG20 can alter tumor growth in an ASS1-proficient environment, similarly to what we observed with ASS1-positive HCT116 cells. Thus, in the absence of rigorous predictive biomarkers that identify synergistic outcomes, it is intriguing to envisage that patient derived xenograft avatars, 3-dimensional organoid cultures or tumor tissue explants^50–52^ could be implemented to predict individual patient’s response and to identify those subjects likely to benefit from ADI-PEG20 and FOLFOX co-treatment. Similarly, the use of arginine deprivation therapy as single treatment remains a promising therapeutic strategy that warrant further investigation in the preclinical and clinical settings.

## Materials and Methods

### TMA Patient cohort

The patient cohort was retrospectively acquired from the Grampian Biorepository (www.biorepository.nhsgrampian.org) and contains tissue samples from 650 patients who had undergone surgery for primary CRC between 1994 and 2009 at Aberdeen Royal Infirmary (Aberdeen, UK). Exclusion criteria included patients who had received neoadjuvant chemotherapy or radiotherapy. The median survival was 103 months (95% CI = 86–120 months), the mean survival was 115 months (95% CI = 108–123 months) and the median follow-up time (“reverse Kaplan-Meier” method) was 88 months (95% CI = 79–97 months). Clinical-pathological characteristics of the patients are summarized in Supplementary Table S1.

TMA also contains 50 normal colon mucosal samples acquired from at least 10 cm distant from the primary cancer TMA and was constructed as previously described^53–55^. All the cases were reviewed and areas of tissue to be sampled were first identified and marked on the appropriate haematoxylin and eosin stained slide by an expert consultant gastro-intestinal pathologist. Two cores each measuring 1 mm in diameter were taken from areas of the corresponding FFPE block and placed in a recipient paraffin block.

The use of human colorectal tissue samples in this study was approved by the Grampian Biorepository scientific access group committee (TR000054). No written consent was required for the use of FFPE tissue samples in the CRC TMA. All animal work was carried out under the PPL# 60/4370 in accordance with the Animals Scientific Procedures Act of 1986. Animal research was approved by the local ethical committee at the University of Leicester.

TMA slides were processed according to standard immunohistochemical protocol described below. Expression levels of ASS1 and OTC were assessed blindfolded using PathXL (Clinical Pathology platform) and the semi quantitative approach of H-Score. Results were validated against automated classification of expression levels using Aperio ImageScope (Leica) and analyzed for agreement using the Cohen’s Kappa coefficient.

### Cell Culture and drugs

All cell lines were maintained in a humidified incubator (37 °C, 5% CO_2_). HCT116 cells were cultured in McCoy’s 5A + GlutaMAX™ + 10% FCS, SW480 and HT29 in Dulbecco’s Modified Eagle’s Medium (DMEM) (4500 mg glucose/L) + GlutaMAX™ + 10% FCS, RKO in Minimum Essential Medium Eagle (MEM) + GlutaMAX™ + 10% FCS. All treatments were in DMEM/F12 (1:1) + GlutaMAX™ and 10% FCS. Cells were exposed to 1 µg/mL ADI-PEG20 (Polaris Pharmaceuticals, San Diego, CA, USA), or 0.5 µg/mL rhArg1peg5000 (Bio-Cancer Treatment International, Hong Kong) or 5 µM of 5-Aza-2′-deoxycytidine (Sigma, UK) or vehicle phosphate-buffered saline (PBS, Thermo-Scientific, UK) control, unless stated otherwise. Oxaliplatin and 5-FU were purchased from Sigma and formulated according to manufacturer’s instructions.

### Cell Proliferation Assays

4000/well cells were seeded in 24-well plates 24 h before treatment. After 24 h cells were counted (day 0) and changed to DMEM/F12 (1:1) (Gibco®, UK) + 10% Dialyzed Fetal Bovine Serum (FBS) (GE Healthcare Life Sciences) containing various concentrations of drugs or arginine. Quadruplicate samples of each concentration were assessed for cell growth by cell counting with the Beckman Z™ Series Coulter Counter. Prior to cell counting, the treatment media was discarded, cells were washed with PBS and trypsinised with 2x Trypsin-EDTA. Trypsin was neutralized with medium and 1 mL of cell suspension was transferred into a Coulter cup with 9 mL of Coulter® Isoton® II diluent (Beckman Coulter, UK). Cells were counted between 8–20 µm.

### Drug Combination studies and evaluation of synergy

Single drug IC_50_ values obtained from corresponding dose-response curves were used for the design of the combination experiments. In a checkerboard 6 × 6 layout; drug concentrations were crossed combined at an equipotency ratio [(IC_50_)_1_/(IC_50_)_2_] to ensure that the observed effects were achieved by equal contribution of both drugs. Cells were counted after 6 days of treatment and data obtained from diagonal constant ratio combinations were analyzed for synergism, additivity or antagonism using the CompuSyn Software (CompuSyn Inc.) according to the Chou and Talalay method^33^.

### EdU Incorporation

10^6^ cells were seeded 24 h before treatment and then changed to medium containing arginine catabolizing agents for 72 h. Prior to FACS analysis cells were pulsed with 10 µM 5-ethyl-2′-deoxyuridine (EdU) for 1 h, harvested according to routine tissue culture protocols and washed with 1% BSA in PBS. After fixation and permeabilization, cells were incubated 15 minutes at room temperature protected from light. Click-iT® Reaction Cocktail was added to cells according to manufacturer’s instructions. Samples were incubated at room temperature for 30 minutes protected from light. Total DNA was stained with 1 µL FxCycle^TM^ Violet stain (Life Technologies, UK) in samples containing 1 mL cell suspension. Samples were incubated for additional 30 minutes on ice in the dark. Samples were analyzed on BD FACSARIA^TM^ II, using 405 nm excitation and 450/50 band-pass emission for total DNA content, whereas a 488 nm excitation with a green emission filter (530/30 nm) was used for detection of EdU with Alexa Fluor® 488 azide. Samples of untreated cells, stained and unstained with EdU or FxCycle^TM^, were also included.

### Antibodies

The antibody against ASS1 was kindly provided by Polaris Pharmaceuticals Inc. Antibodies targeting Cyclin D1 (DCS6 and 92G2) and Cyclin D3 (DCS22), total (53H11) total and phospho (Thr37/46) (236B4) 4E-BP1, total (5G10) and phospho (Ser235/236) (D57.2.2E) ribosomal protein S6, and total/cleaved-PARP (46D11) were from Cell Signalling Technology (Leiden, The Netherlands). Actin (C-11) primary antibody and secondary mouse (sc-2005), rabbit (sc-2030) and goat (sc-2020) antibodies were purchased from Santa Cruz. OTC (ab91418) and Ki67 (ab833) antibodies were from Abcam (Cambridge, MA, USA). All primary antibodies for western blotting were diluted at in 3% BSA at a working concentration 1:1,000 overnight. All secondary antibodies were diluted at 1:10,000 in 3% milk.

### Western-blot

Whole-cell lysates of CRC cell lines and xenograft tissues were prepared in Complete Lysis-M buffer (Roche, Germany). Protein quantification was performed using the Pierce™ BCA protein assay (Thermo Fisher Scientific, UK). 30 µg of total protein per lane was loaded on acrylamide gel. Following the SDS-PAGE electrophoresis, proteins were transferred to a nitrocellulose membrane (Geneflow, UK), blocked for 1 h in 5% milk and incubated overnight with primary antibodies at 4 °C. Membranes were then washed with PBS-0,01%Tween (SIGMA, UK) and incubated with appropriate secondary antibodies at room temperature for 1 h. Proteins were visualized using the Enhanced Chemiluminescence Luminol (ECL) reagent (Geneflow Ltd., UK). Images were captured using the GeneGnome XRQ system (Syngene, UK) or Kodak X-Ray films. The protein band intensity was measured using ImageJ software and the relative expression levels of the proteins were normalized against actin. The protein band intensity was measured using densitometry performed with ImageJ software. Proteins presented as double bands were selected and quantified as a whole. The relative expression levels of the proteins were normalized against actin.

### Immunohistochemistry (IHC)

The immunostaining was performed using Novolink^TM^ Polymer Detection System (Leica Biosystems, UK). Following deparaffinization, rehydrated sections were boiled for 20 minutes in antigen retrieval buffer (1X Tris-EDTA, pH 9). Endogenous peroxidase activity was neutralized using peroxidase block for 5 minutes. Sections were then blocked in protein block buffer for 30 minutes and incubated with primary antibodies for 2 h at room temperature. Peroxidase chromogenic reaction was developed with DAB working solution according to manufacturer’s instructions. Slides were counterstained with haematoxylin and mounted with DPX mounting media and scanned with Hamamatsu NanoZoomer-XR Digital scanner. Staining was evaluated with Aperio ImageScope software (Leica) and the semi-quantitative H-score calculated using the following formula:

$$[1{\rm{x}}\,( \% \,{\rm{weak}}\,{\rm{positive}}\,{\rm{cells}})+2{\rm{x}}\,( \% \,{\rm{positive}}\,{\rm{cells}})+3{\rm{x}}\,( \% \,{\rm{strong}}\,{\rm{positive}}\,{\rm{cells}})].$$

### *In vivo* xenograft studies

8-week-old, female Foxn1^nu^ mice were purchased from Charles River, UK. Mice were kept in ventilated cages exposed to 12 h light/dark cycles under pathogen free conditions. Prior to xenografting, HCT116-luc2 (Perkin Elmer), RKO and SW480 cells were harvested at 70–80% confluency and resuspended to a final concentration of 10^7^ cells per 1 ml Matrigel (BD Biosciences):Serum Free medium [1:1]. 10^6^ cells were then injected subcutaneously unto the flank of each animal. A week after, mice with established tumors were randomized and treated with ADI-PEG20 5IU/animal once a week or rhArg1peg5000 5 mg/animal twice a week or vehicle control (PBS). Mice bearing HCT116 tumors were randomized to arginine-free diet (57M7) or control diet (5CC7) (TestDiet®) right after the initial cell injections. Body weight and tumor size were measured weekly. Further to the manual measurements, mice bearing HCT-116 luc2 tumors were monitored via the IVIS Spectrum Preclinical Imaging System (PerkinElmer®) once a week. Prior to *in vivo* imaging mice were administered subcutaneously with 150 mg/kg Xenolight RediJect D-Luciferin (PerkinElmer®), anesthetised and imaged with IVIS 10 minutes post injection. When tumors reached 17 mm diameter animals were sacrificed under terminal anesthesia (3–5% Isoflurane). Blood was collected by cardiac puncture. Tumors were excised, weighted, and fixed in formalin or snap-frozen. Study groups were not based on power calculations and experimenters were not blinded to the randomly allocated treatment groups.

### Statistical analysis

The error bars represent the ±Standard Error of the Mean (±SEM). Statistical significance between two groups was determined by two-tailed unpaired Student’s t-test, whereas multiple One-Way Anova analysis was conducted to compare controls against multiple drug concentrations (Prism version 7.0, GraphPad Software, Inc.). The animal xenograft studies were assessed for statistical significance using the mixed linear regression model in Stata software (StataCorp LP, College Station, TX, USA). Statistical analysis of TMA cohort patient data including the Mann-Whitney U test, Wilcoxon signed rank test, chi-squared test, and Cox multi-variate analysis (variables entered as categorical variables) was performed using IBM SPSS version 24 for Windows 7^TM^ (IBM, UK).

## Electronic supplementary material

Supplemental data
