# Supplementary material for: Sensitivity of Colorectal Cancer to Arginine Deprivation Therapy is Shaped by Differential Expression of Urea Cycle Enzymes
Source: Sci Rep. 2018 Aug 14;8:12096. doi: 10.1038/s41598-018-30591-7 (PMC6092409; doi:10.1038/s41598-018-30591-7)
Supplement: Supplementary file 1 — Supplemental data [file 41598_2018_30591_MOESM1_ESM.pdf]

Supplementary Information

Sensitivity of Colorectal Cancer to Arginine Deprivation Therapy is Shaped by Differential Expression of Urea Cycle Enzymes.

Constantinos Alexandrou, Saif Sattar Al-Aqbi, Jennifer A Higgins, William Boyle, Ankur Karmokar, Catherine Andreadi, Jin-Li Luo, David A Moore, Maria Viskaduraki, Matthew Blades, Graeme I Murray, Lynne M Howells, Anne Thomas, Karen Brown, Paul N Cheng and Alessandro Rufini.

Supplementary Figure Legends

Supplementary Figure S1. A) Schematic of the urea cycle. Enzymes are in red and show the role of ASS1 and OTC in recycling citrulline and ornithine respectively. ASS1 argininosuccinate synthase 1; ASL, argininosuccinate lyase; OTC, ornithine transcarbamylase; CPS1, carmaboylphosphate synthase 1, ARG1, arginase 1. **B)** RKO cells were grown in arginine-free medium for 5 days. At day 5 cell culture medium was replenished with arginine (147.5 mg/L) and cells were counted for six additional days in the presence of arginine. Data are plotted as mean ± SEM.

Supplementary Figure S2. Western blot analysis of the mTOR downstream target 4E-BP1 and ribosomal S6 proteins in **A)** HCT116, **B)** RKO, **C)** SW480 and **D)** HT29 CRC cell lines following arginine deprivation for the indicated times. Both phosphorylated and total levels of the proteins are reported. Actin was used as endogenous loading control. Original western blots are reported in Supplementary Figure S15.

Supplementary Figure S3. Representative sequential images from individual mice fed with Control and Arginine-free diet over time. HCT116-Luc2 tumor xenografts were imaged at the indicated days. Prior to imaging in the IVIS imaging chamber, mice were injected subcutaneously with RediJect D-Luciferin (150 mg/kg).

Supplementary Figure S4. A) ASS1 and **B)** OTC immunohistochemical (DAB) staining scoring of representative TMA, utilizing the semi quantitative approach of H-score (0= negative, 1-100 = weak positive, 101-200 = positive and 201-300 strong positive) as obtained by automated software classification (right) and manual scoring (left). **C)** Cohen's Interobserver agreement between A) and B): kappa coefficient analysis kappa values between 0.41-0.60 indicate moderate agreement whereas values from 0.61-080 indicate substantial agreement.

Supplementary Figure S5. A) Meta-analysis of OTC expression in CRC datasets performed using the online tool CancerMA (P<0.001). The Forest plot visualizes the meta-analysis results for *OTC* gene expression in the indicated colorectal cancer

datasets. Each dataset is illustrated by a square; the position on the x-axis representing the measure estimate of *OTC* gene expression (expressed as logarithm of “fold over control tissue”, lg2FC ratio), the size of the square being proportional to the weight of the study, and the horizontal line through it reflecting the confidence interval of the estimate. The diamond sign indicates the calculated average *OTC* lg2FC ratio and its confidence interval. OTC was found to be highly downregulated in all datasets analyzed. Colorectal_Ad, Colorectal adenoma; Colorectal_ADC, Colorectal adenocarcinoma; Colorectal_C, Colorectal cancer; Colorectal_Ep_C, Colorectal carcinoma, epithelial cells; Colorectal_Met, Colorectal cancer, metastatic; Colorectal_MRC, Colorectal cancer, metastatic recurrence; Colorectal_Muc_Ad, Colorectal adenoma, mucosa; Colorectal_Muc_C, Colorectal carcinoma, mucosa; Colorectal_PreAd, Precancerous adenoma of the colon. **B)** Table showing the downregulation of OTC expression (FC= fold over normal tissue control) and associated P values in the indicated dataset performed using the online tool Oncomine.

Supplementary Figure S6. A) Western blot analysis of ASS1 and c-Myc expression in the indicated cell lines treated with vehicle control or ADI-PEG20 (1 μg/mL) and rhArg1peg5000 (0.5 μg/mL) for the indicated times. Original western blots are reported in Supplementary Figure S15. **B)** and **C)** show linear correlation analysis of ASS1 and c-Myc expression levels in ADI-PEG20 and rhArg1peg5000 treated cells, respectively.

Supplementary Figure S7. Western blot analysis of protein expression of the urea cycle enzyme OTC in cells treated for the indicated time with rhArg1peg5000 (0.5 μg/mL). Actin was used as endogenous loading control. Original western blots are reported in Supplementary Figure S15.

Supplementary Figure S8. A) Western blot assessment of the apoptotic markers cleaved-PARP in CRC cell lines treated with rhArg1peg5000 for the indicated times. Original western blots are reported in Supplementary Figure S15. **B)** Quantification of cleaved PARP from three independent experiments, two-way ANOVA, *P<0.05; **P<0.01; ns=P>0.05. Data were normalized to actin loading control. Etoposide was used as positive control for apoptosis induction.

Supplementary Figure S9. Western blot analysis of mTOR pathway in the indicated cell lines. Cells were treated with vehicle control or rhArg1peg5000 (0.5 μg/mL).

Arginase effect was assessed in short (0h to 8h) and long incubation intervals (24h to 72h), detecting the phosphorylation status of the mTOR downstream targets 4E-BP1 and ribosomal S6 protein. Original western blots are reported in Supplementary Figure S15.

Supplementary Figure S10. Endpoint body weight of mice xenografted with **A)** RKO or **B)** SW480 cells and treated with vehicle control or rhArg1peg5000. Each dot represents one animal, mean ± SEM are also plotted. Data were analyzed using non-parametric Mann-Whitney test, n=8, ns = P>0.05.

Supplementary Figure S11. Western blot analysis of mTOR pathway in the indicated cell lines. Cells were treated with vehicle control or ADI-PEG20 (1 μg/mL) and assessed at the indicated time points to detect the phosphorylation status of the mTOR downstream targets 4E-BP1 and ribosomal S6 protein. Original western blots are reported in Supplementary Figure S15.

Supplementary Figure S12. A) Time course measurement of body weight of mice treated with vehicle control or ADI-PEG20. Each data point represent mean ± SEM. Data were analyzed with two-way ANOVA and Sidak's multiple comparison tests. *P<0.05. **B)** Endpoint body weight of mice xenografted with RKO cells and treated with vehicle control or ADI-PEG20. Each dot represents one animal, mean ± SEM are also plotted. Data were analyzed using non-parametric Mann-Whitney test, n=8, **P<0.01.

Supplementary Figure S13. Isobolograms obtained using the Chou-Talalay methods for combination studies of with ADI-PEG20 and 5-FU or oxaliplatin. Drugs were used at the indicated range of concentrations. The effect of treatment combination is shown over a range of ICs (IC_{50} to IC_{95}). The colored lines connect the IC_s values for the single drugs, the combinatorial concentrations for the equivalent ICs are shown as indicated in the legend. When the combinatorial concentration falls in proximity of the ICs line the two drugs act additively (CI~1); combinations above and below the green indicates antagonism (CI>1) and synergism (CI<1), respectively. Data were analyzed using CompuSyn software.

Supplemental Figure S14. Isobolograms obtained using the Chou-Talalay methods for combination studies of with rhArg1peg5000 and 5-FU or oxaliplatin. Drugs were used at the indicated range of concentrations. The effect of treatment combination is shown over a range of ICs (IC_{50} to IC_{95}). The colored lines connect the IC_s values for

the single drugs, the combinatorial concentrations for the equivalent ICs are shown as indicated in the legend. When the combinatorial concentration falls in proximity of the ICs line the two drugs act additively (CI~1); combinations above and below the green indicates antagonism (CI>1) and synergism (CI<1), respectively. Data were analyzed using CompuSyn software.

Supplemental Figure S15. Original, uncropped western blots images corresponding to the cropped blots for the indicated figures.

Figure S1

A

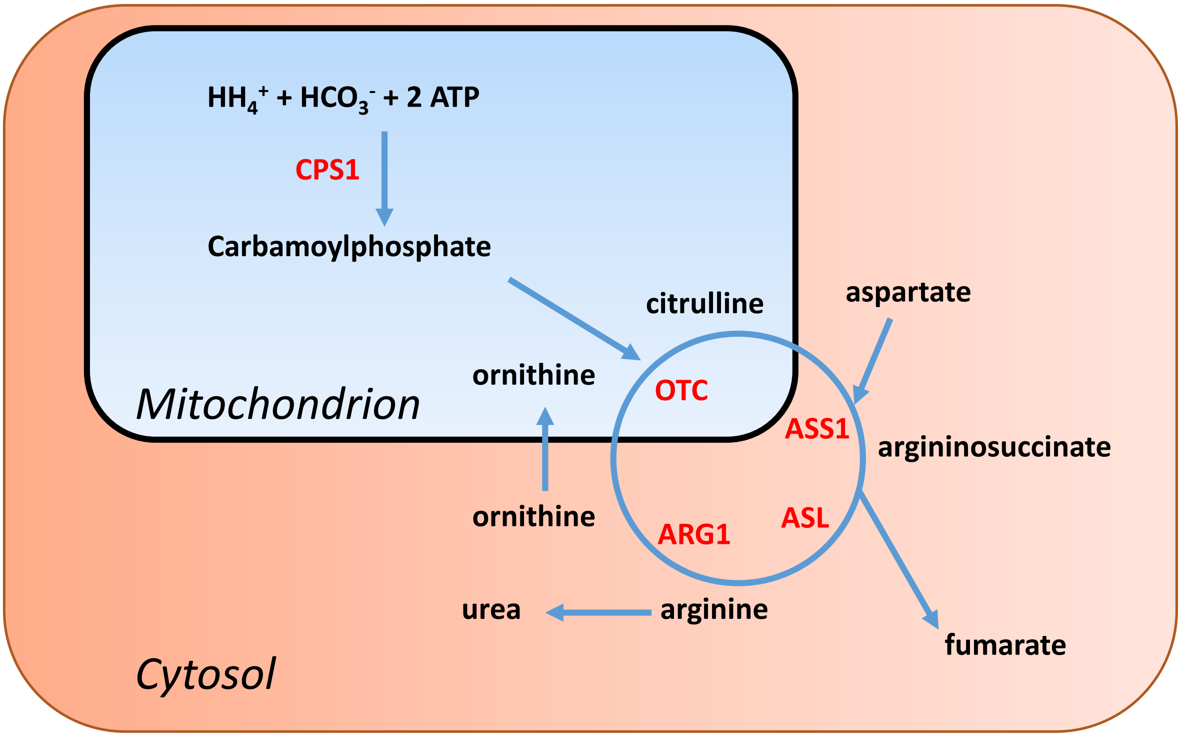
HH$_4^+$ + HCO$_3^-$ + 2 ATP
CPS1
Carbamoylphosphate
citrulline
aspartate
ornithine
OTC
Mitochondrion
ASS1
argininosuccinate
ornithine
ASL
ARG1
urea
arginine
fumarate
Cytosol

B

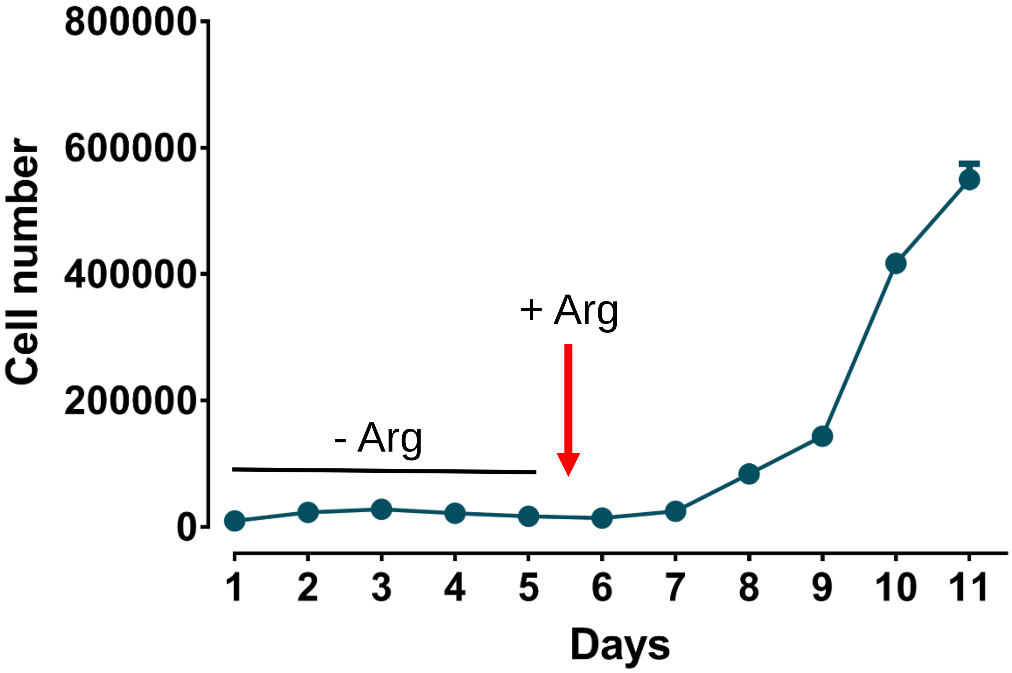
Cell number
800000
600000
400000
200000
0
+ Arg
- Arg
1
2
3
4
5
6
7
8
9
10
11
Days

Figure S2

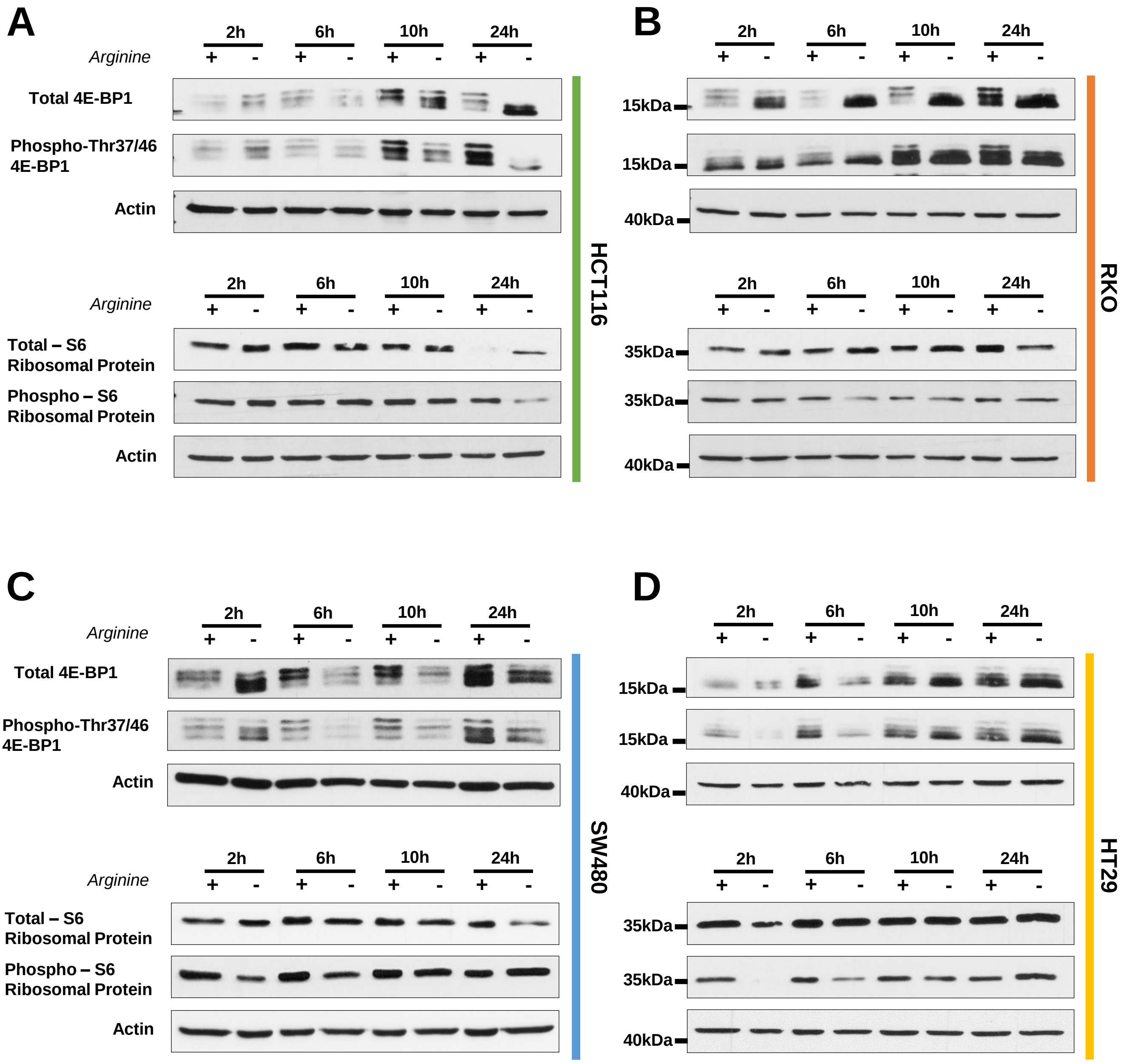
A
B
C
D
2h
6h
10h
24h
Arginine
+
-
Total 4E-BP1
Phospho-Thr37/46 4E-BP1
Actin
Total – S6 Ribosomal Protein
Phospho – S6 Ribosomal Protein
15kDa
35kDa
40kDa
HCT116
RKO
SW480
HT29

Figure S3

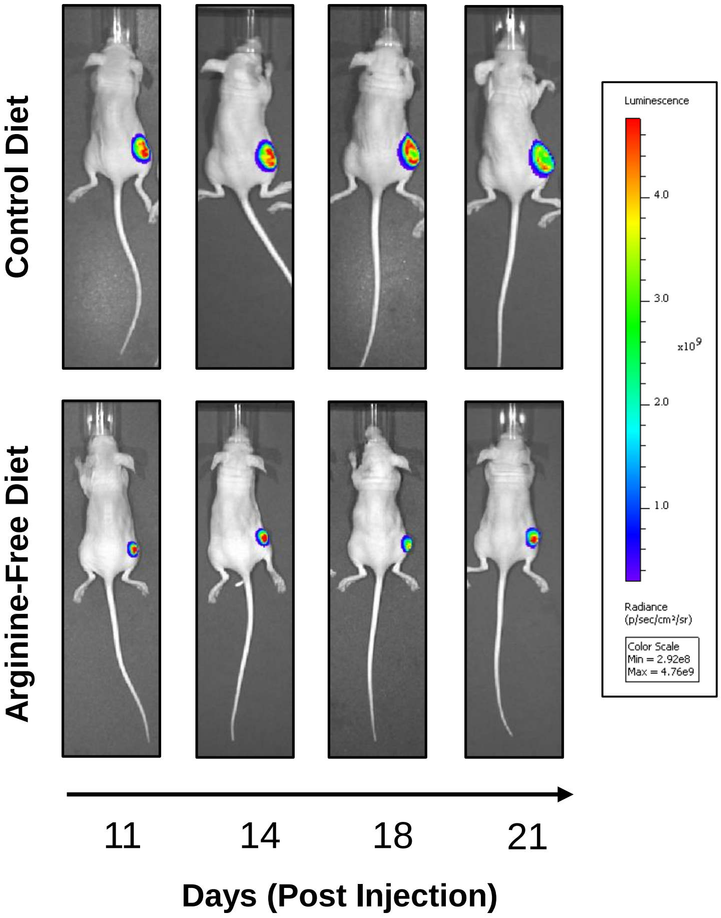
Control Diet
Arginine-Free Diet
Luminescence
4.0
3.0
x10^9
2.0
1.0
Radiance
(p/sec/cm²/sr)
Color Scale
Min = 2.92e8
Max = 4.76e9
11
14
18
21
Days (Post Injection)

Figure S4

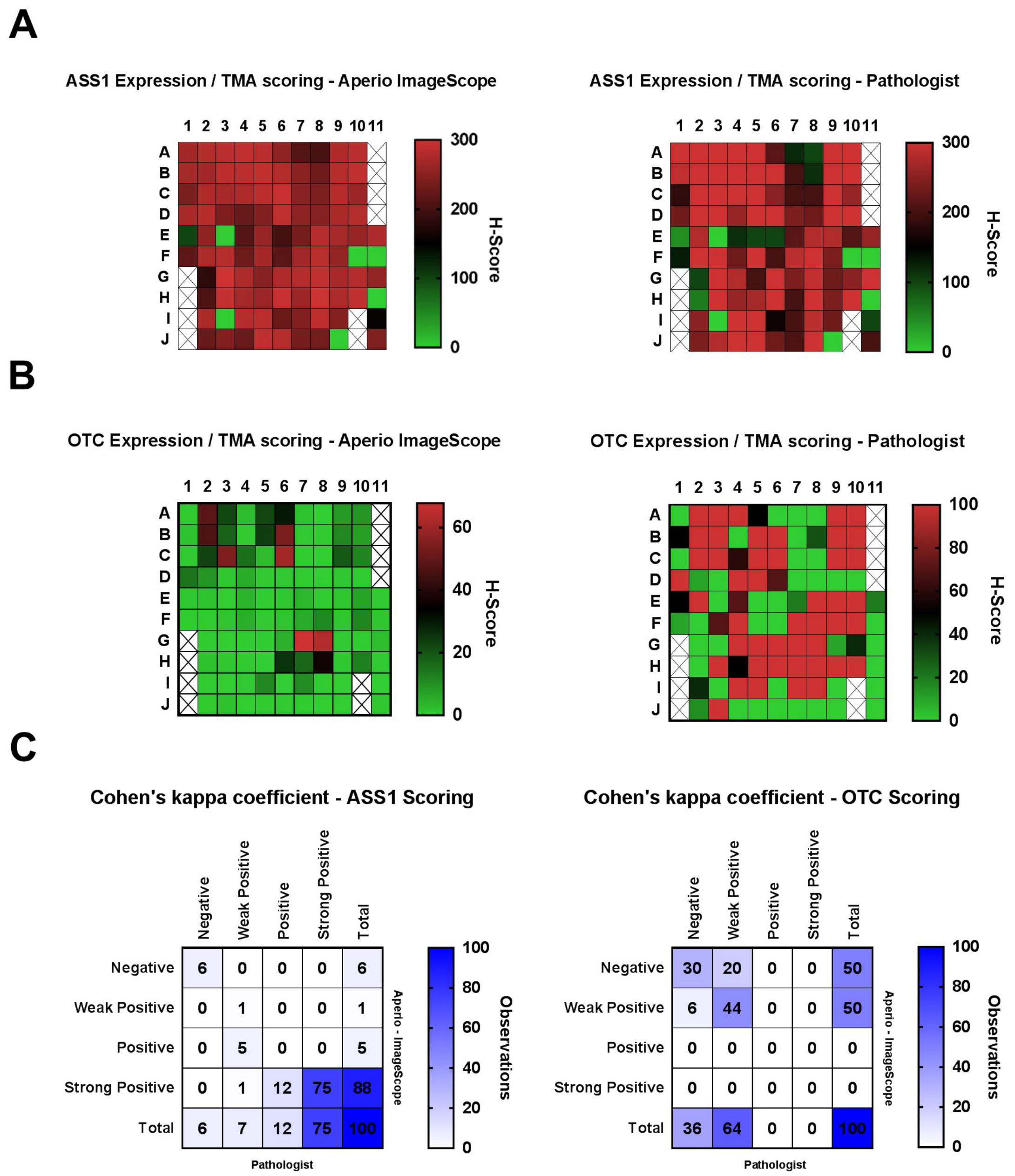

Kappa = 0.454

Strenght of agreement : **moderate**

Weighted kappa = 0.686

Strenght of agreement : **good**

Kappa = 0.480

Strenght of agreement : **moderate**

Figure S5

A

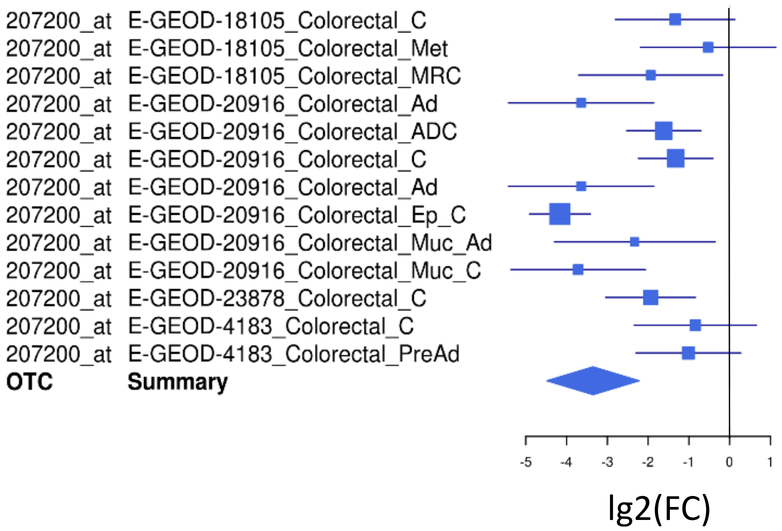

B

Dataset	FC	P value
Hong	-9.176	6.16E-9
TCGA	-7.730	2.93E-23
Ki	-3.082	1.57E-5
Kaiser	-1.899	7.48E-8
Gaedcke	-1.661	1.12E-5

Figure S6

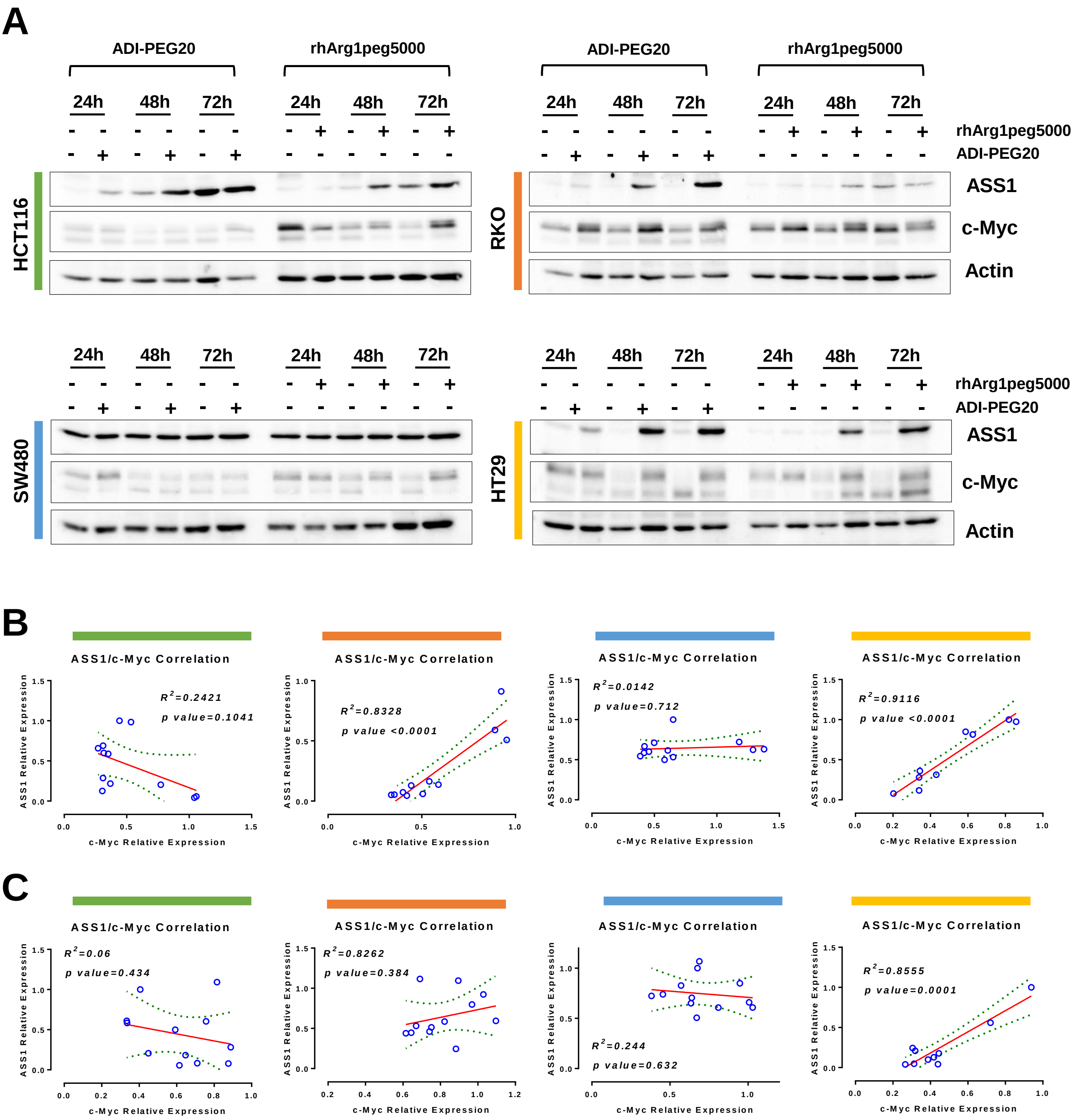
A
ADI-PEG20
rhArg1peg5000
24h
48h
72h
rhArg1peg5000
ADI-PEG20
HCT116
RKO
SW480
HT29
ASS1
c-Myc
Actin
B
ASS1/c-Myc Correlation
ASS1 Relative Expression
c-Myc Relative Expression
R^2=0.2421
p value=0.1041
R^2=0.8328
p value <0.0001
R^2=0.0142
p value=0.712
R^2=0.9116
p value <0.0001
C
ASS1/c-Myc Correlation
ASS1 Relative Expression
c-Myc Relative Expression
R^2=0.06
p value=0.434
R^2=0.8262
p value=0.384
R^2=0.244
p value=0.632
R^2=0.8555
p value=0.0001

Figure S7

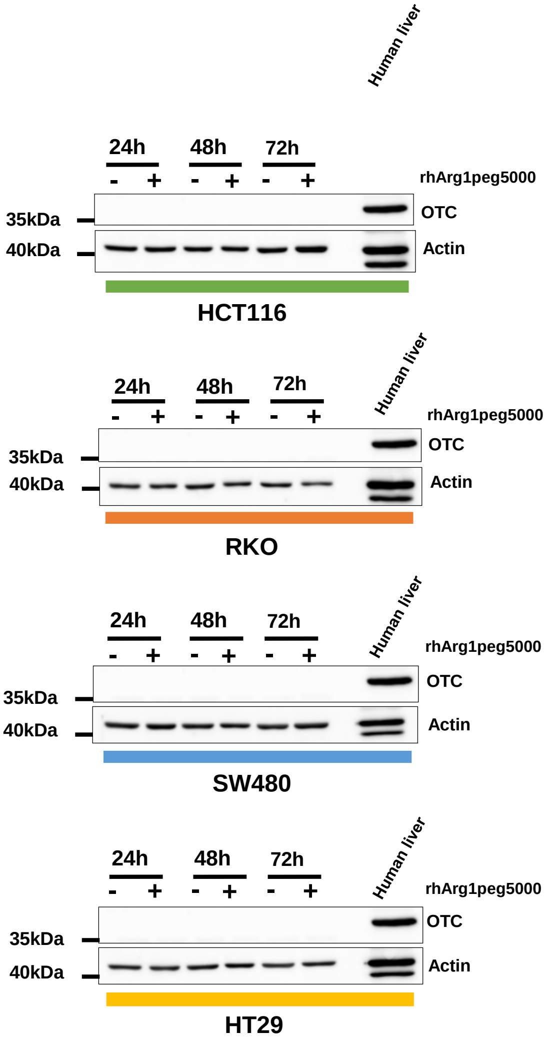

Human liver
24h
48h
72h
- + - + - +
rhArg1peg5000
35kDa
OTC
40kDa
Actin
HCT116
Human liver
24h
48h
72h
- + - + - +
rhArg1peg5000
35kDa
OTC
40kDa
Actin
RKO
Human liver
24h
48h
72h
- + - + - +
rhArg1peg5000
OTC
35kDa
Actin
40kDa
SW480
Human liver
24h
48h
72h
- + - + - +
rhArg1peg5000
OTC
35kDa
Actin
40kDa
HT29

Figure S8

A

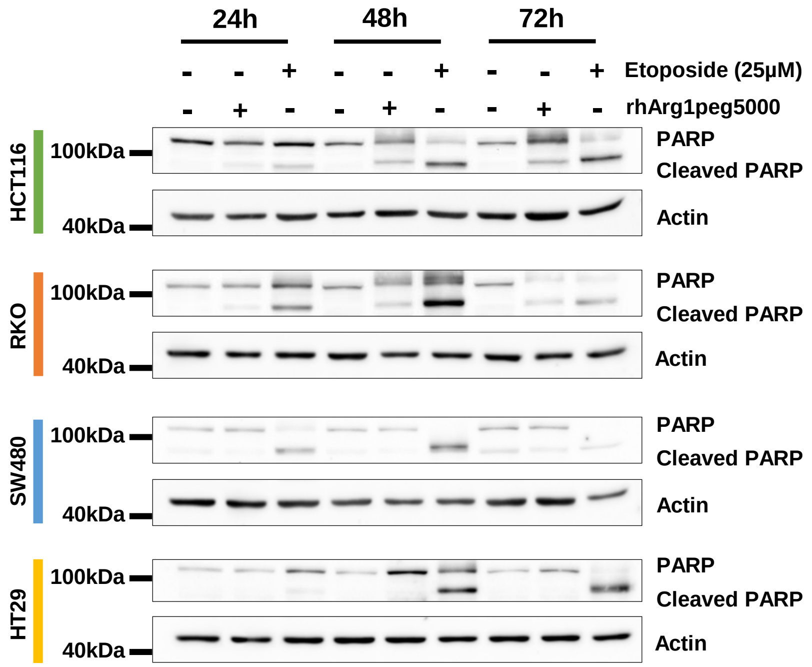
24h
48h
72h
Etoposide (25μM)
rhArg1peg5000
HCT116
RKO
SW480
HT29
100kDa
40kDa
PARP
Cleaved PARP
Actin

B

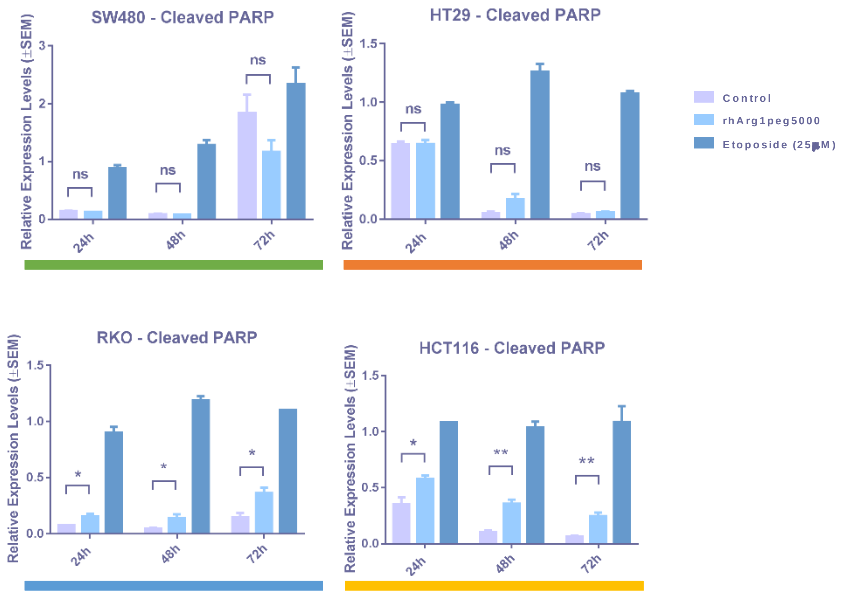
SW480 - Cleaved PARP
HT29 - Cleaved PARP
RKO - Cleaved PARP
HCT116 - Cleaved PARP
Relative Expression Levels (±SEM)
ns
*
**
24h
48h
72h
Control
rhArg1peg5000
Etoposide (25μM)

Figure S9

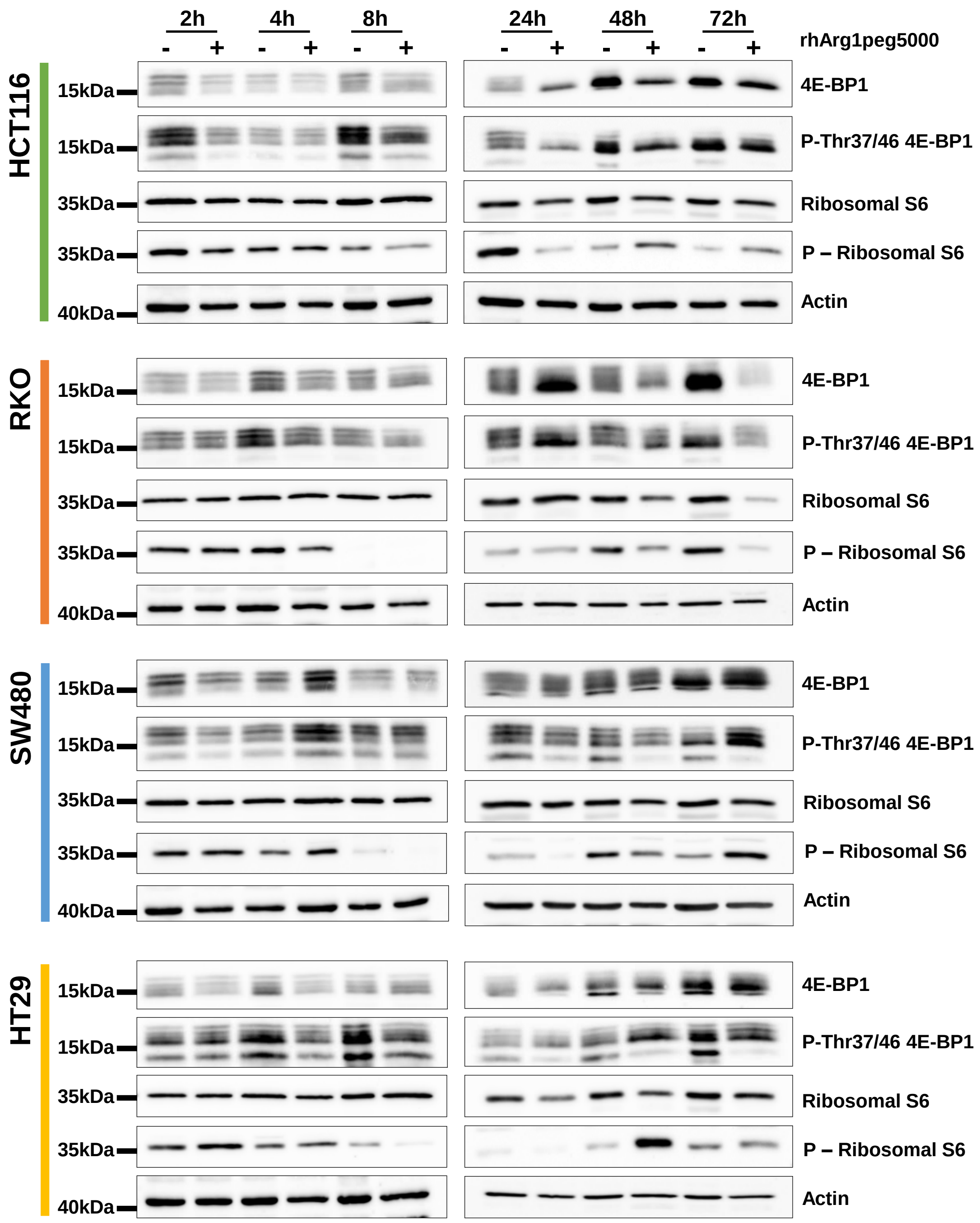
2h
4h
8h
24h
48h
72h
- + - + - +
- + - + - +
rhArg1peg5000
HCT116
RKO
SW480
HT29
15kDa
35kDa
40kDa
4E-BP1
P-Thr37/46 4E-BP1
Ribosomal S6
P – Ribosomal S6
Actin

Figure S10

A

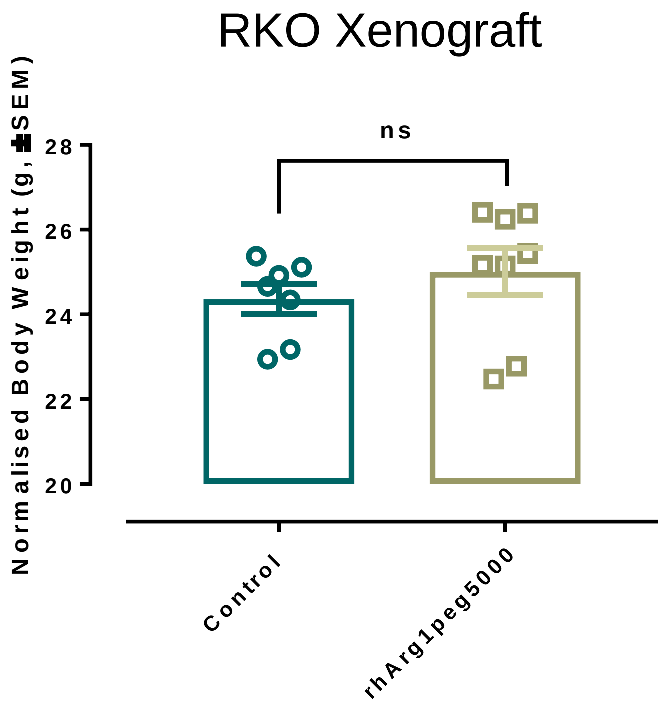

B

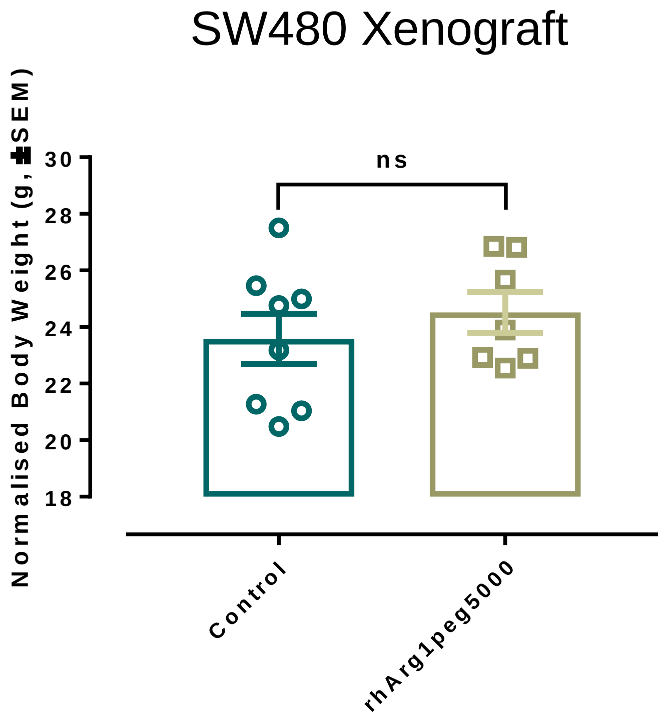

Figure S11

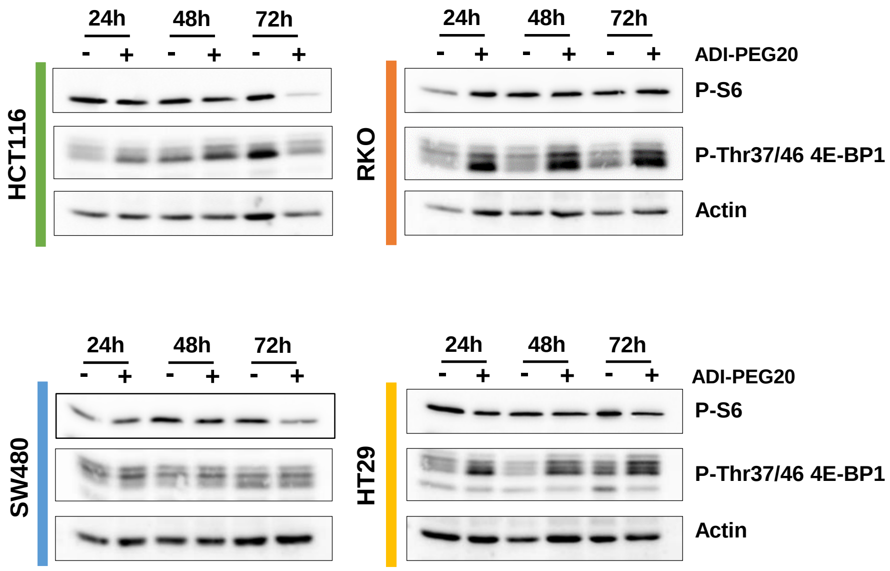
HCT116
RKO
SW480
HT29
24h
48h
72h
ADI-PEG20
P-S6
P-Thr37/46 4E-BP1
Actin

Figure S12

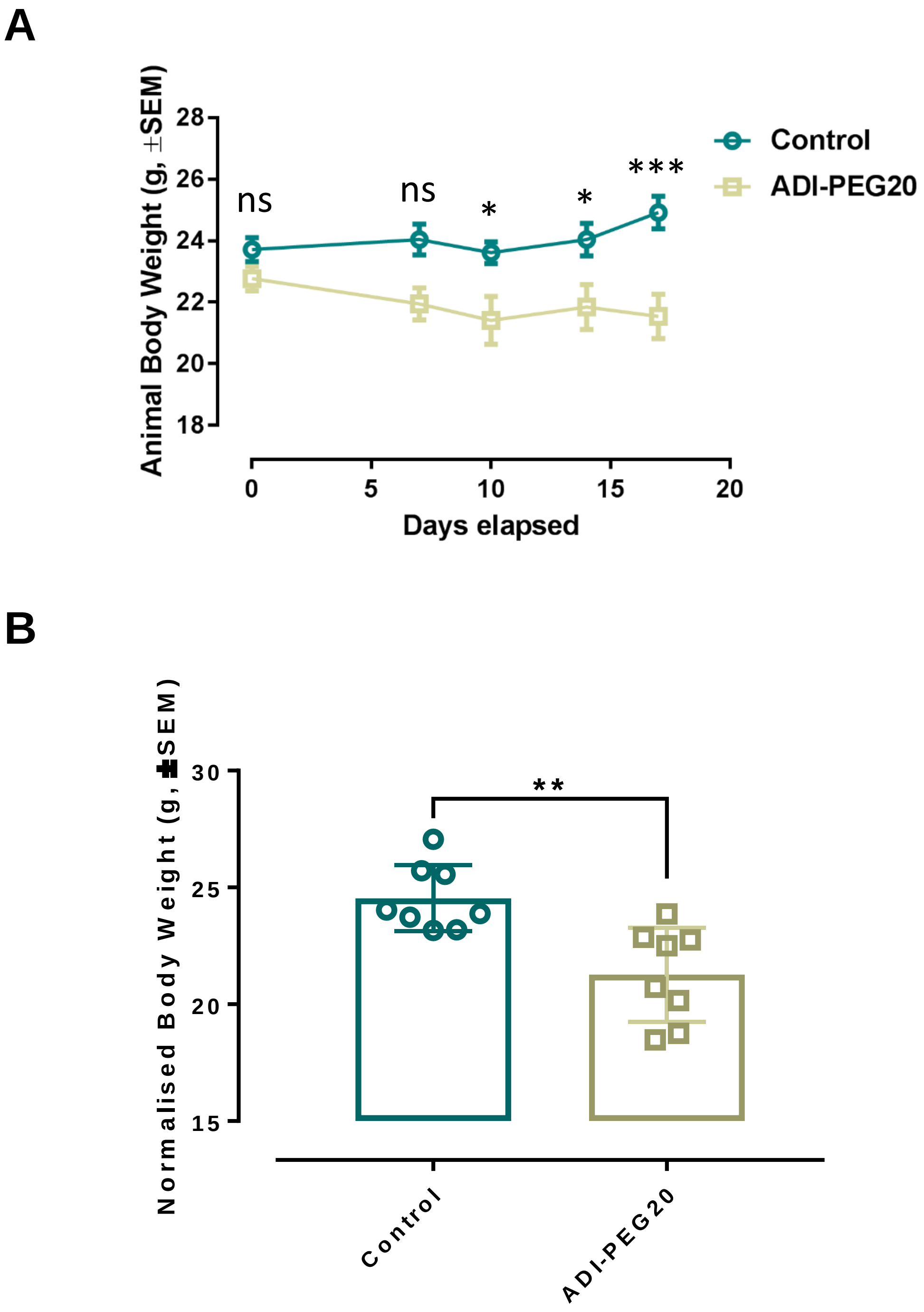
A
Animal Body Weight (g, ±SEM)
28
26
24
22
20
18
ns
ns
*
*
***
Control
ADI-PEG20
0
5
10
15
20
Days elapsed
B
Normalised Body Weight (g, ±SEM)
30
25
20
15
**
Control
ADI-PEG20

Figure S13

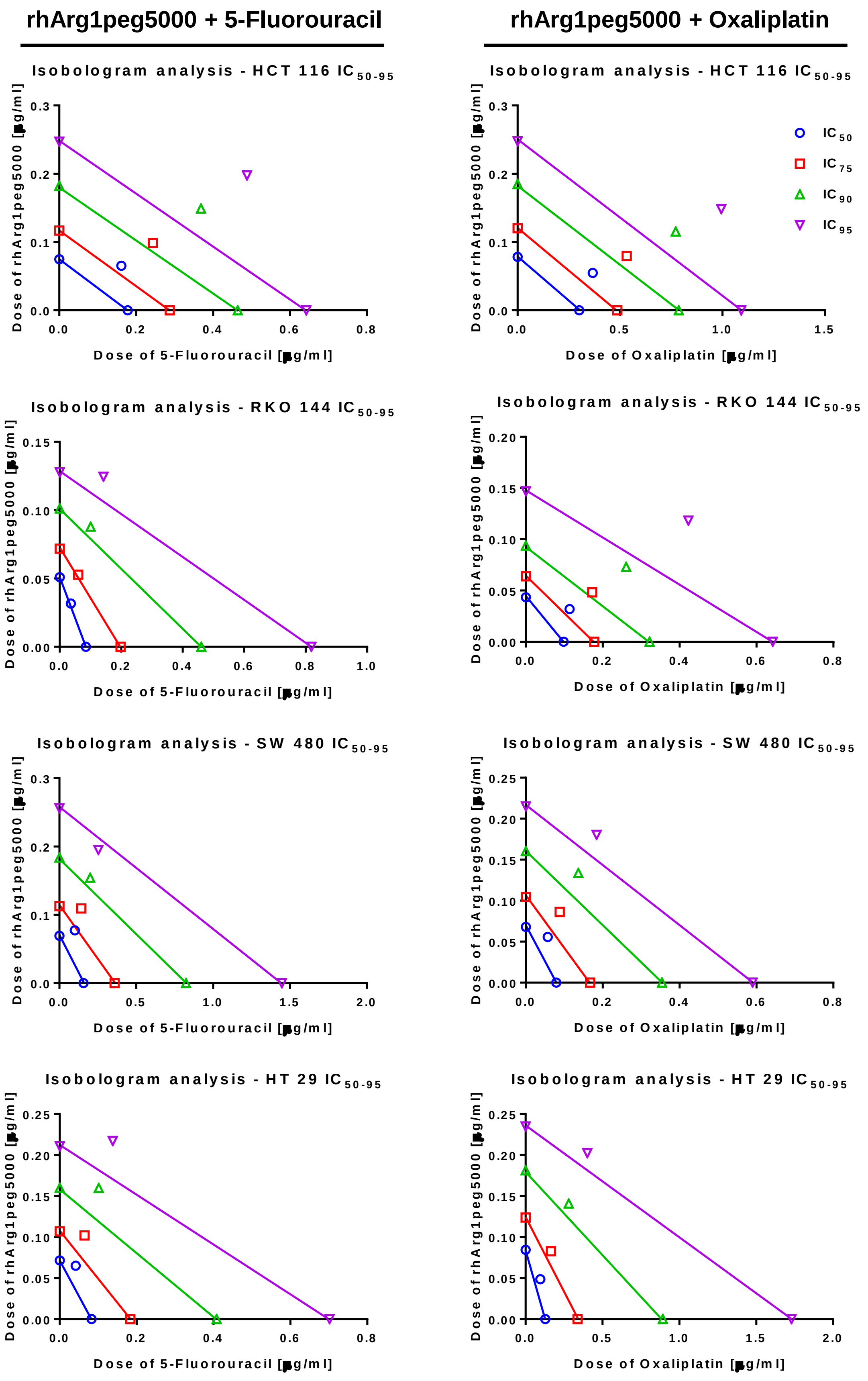
rhArg1peg5000 + 5-Fluorouracil
rhArg1peg5000 + Oxaliplatin
Isobologram analysis - HCT 116 IC_{50-95}
Isobologram analysis - HCT 116 IC_{50-95}
IC_{50}
IC_{75}
IC_{90}
IC_{95}
Isobologram analysis - RKO 144 IC_{50-95}
Isobologram analysis - RKO 144 IC_{50-95}
Isobologram analysis - SW 480 IC_{50-95}
Isobologram analysis - SW 480 IC_{50-95}
Isobologram analysis - HT 29 IC_{50-95}
Isobologram analysis - HT 29 IC_{50-95}
Dose of rhArg1peg5000 [µg/ml]
Dose of 5-Fluorouracil [µg/ml]
Dose of Oxaliplatin [µg/ml]

Figure S14

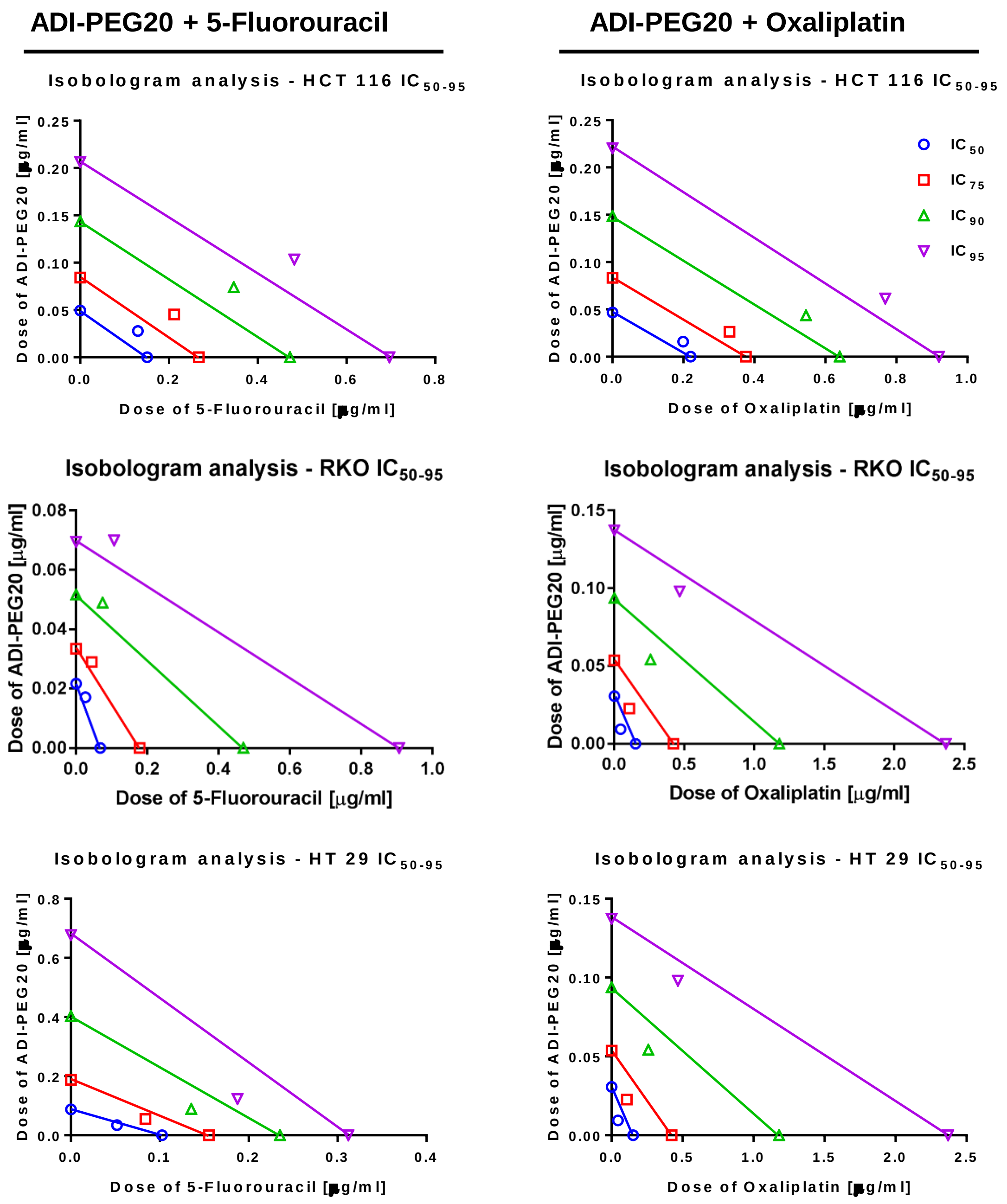
ADI-PEG20 + 5-Fluorouracil
ADI-PEG20 + Oxaliplatin
Isobologram analysis - HCT 116 IC50-95
Dose of ADI-PEG20 [µg/ml]
Dose of 5-Fluorouracil [µg/ml]
Isobologram analysis - HCT 116 IC50-95
IC50
IC75
IC90
IC95
Dose of ADI-PEG20 [µg/ml]
Dose of Oxaliplatin [µg/ml]
Isobologram analysis - RKO IC50-95
Dose of ADI-PEG20 [µg/ml]
Dose of 5-Fluorouracil [µg/ml]
Isobologram analysis - RKO IC50-95
Dose of ADI-PEG20 [µg/ml]
Dose of Oxaliplatin [µg/ml]
Isobologram analysis - HT 29 IC50-95
Dose of ADI-PEG20 [µg/ml]
Dose of 5-Fluorouracil [µg/ml]
Isobologram analysis - HT 29 IC50-95
Dose of ADI-PEG20 [µg/ml]
Dose of Oxaliplatin [µg/ml]

Figure S15

Western blots relative to Figure 1

Cyclin D1 **Actin**

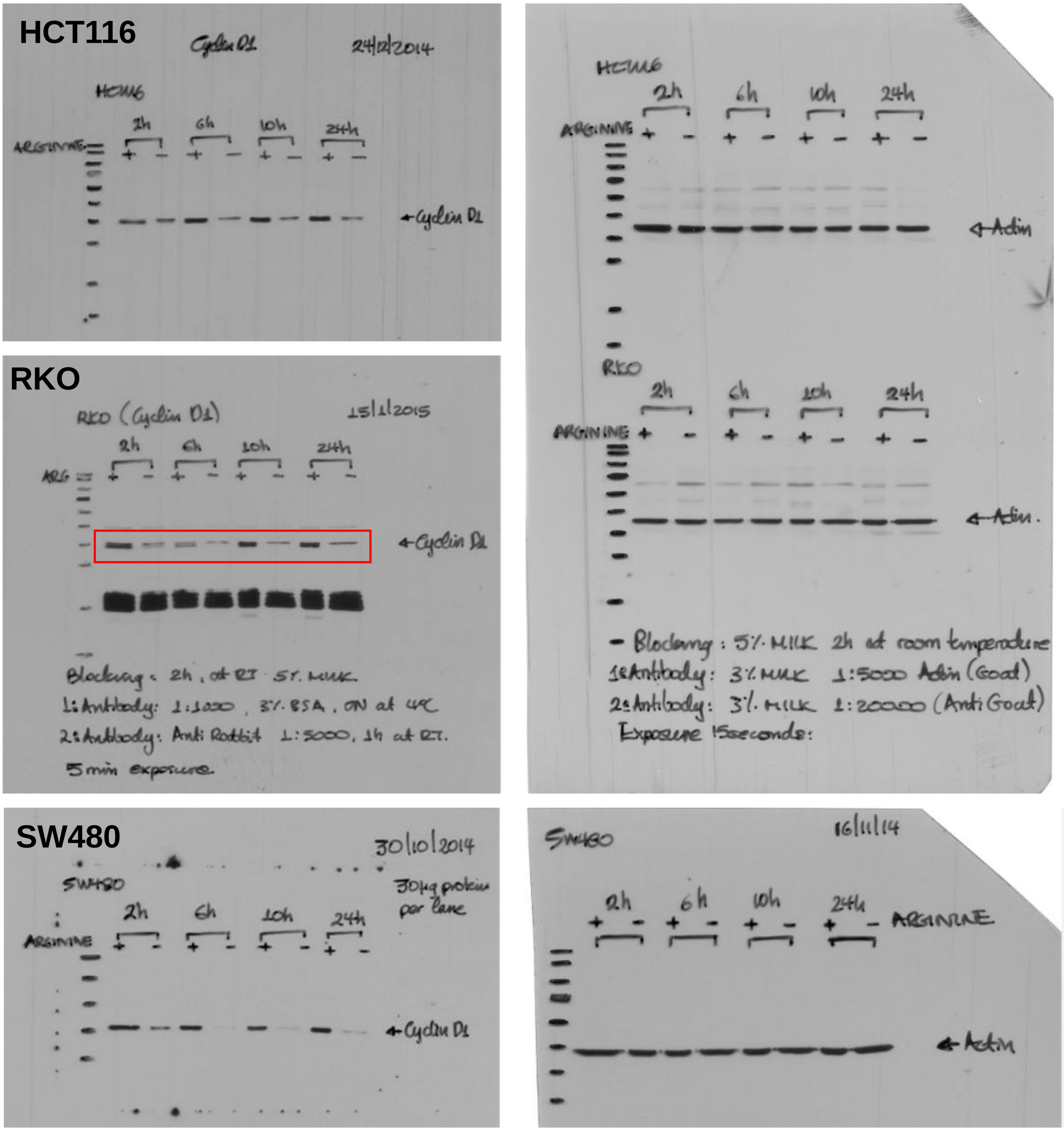

Figure S15

Western blots relative to Figure 2

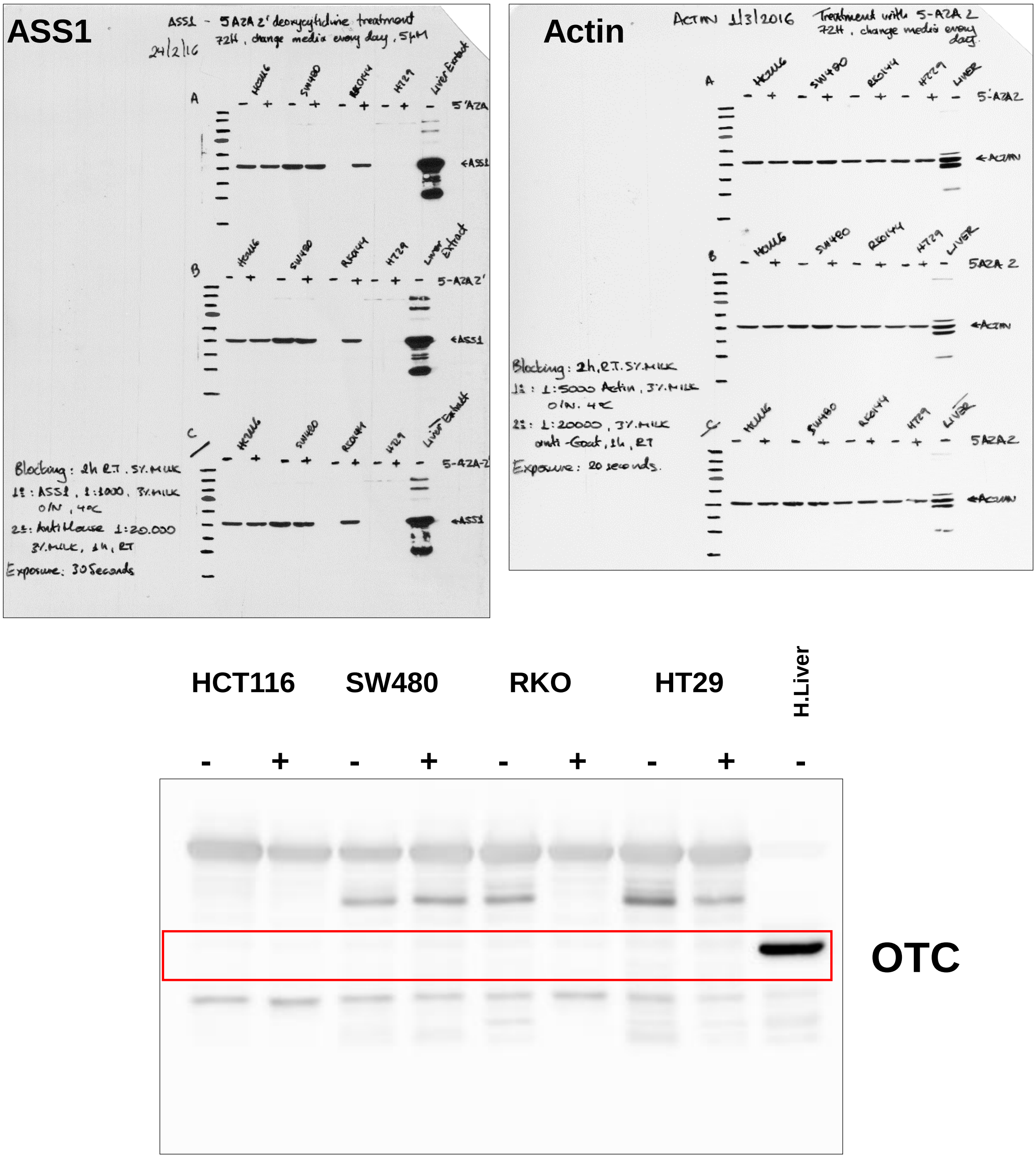

Figure S15

Western blots relative to Figure 3

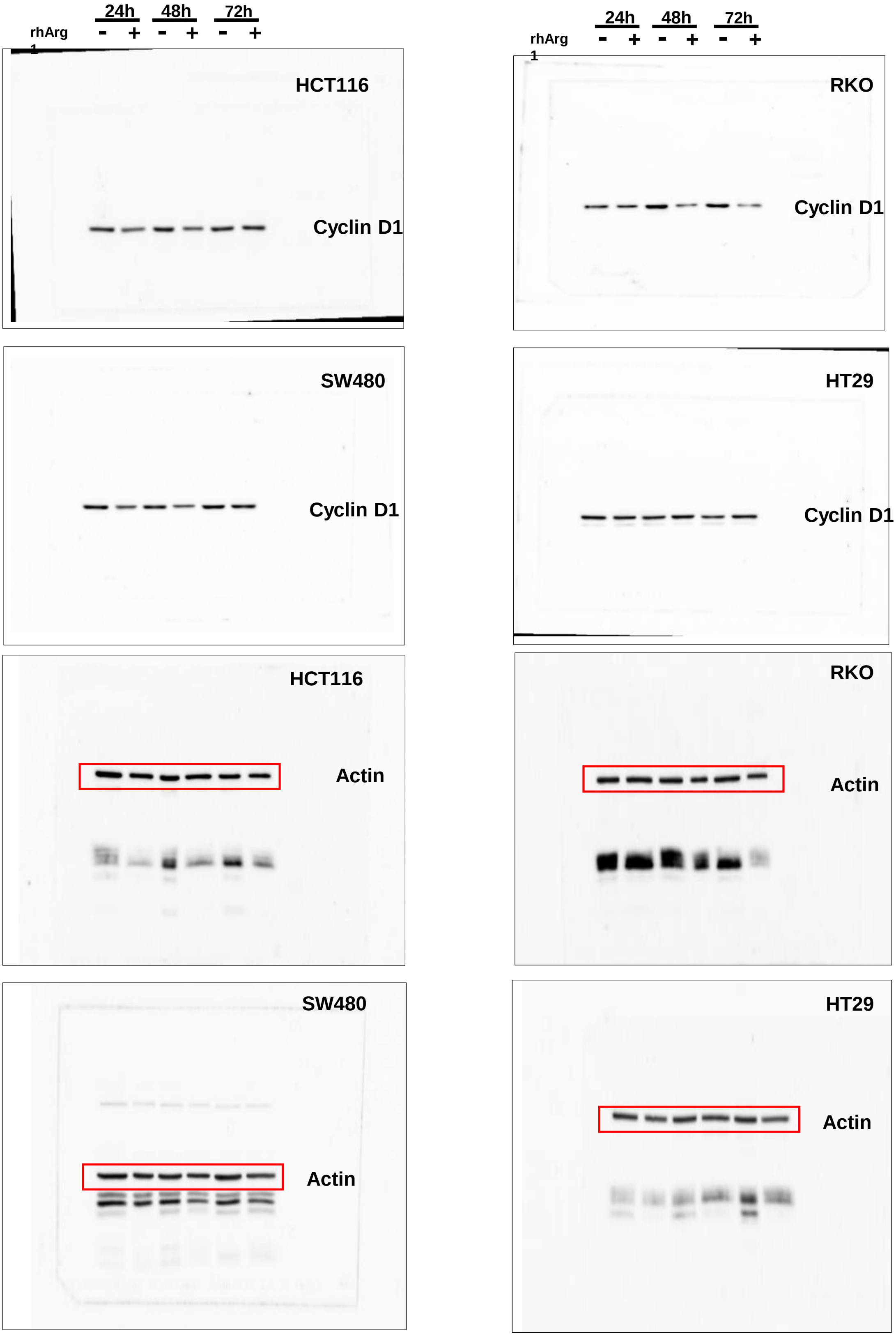

Figure S15

Western blots relative to Figure 3

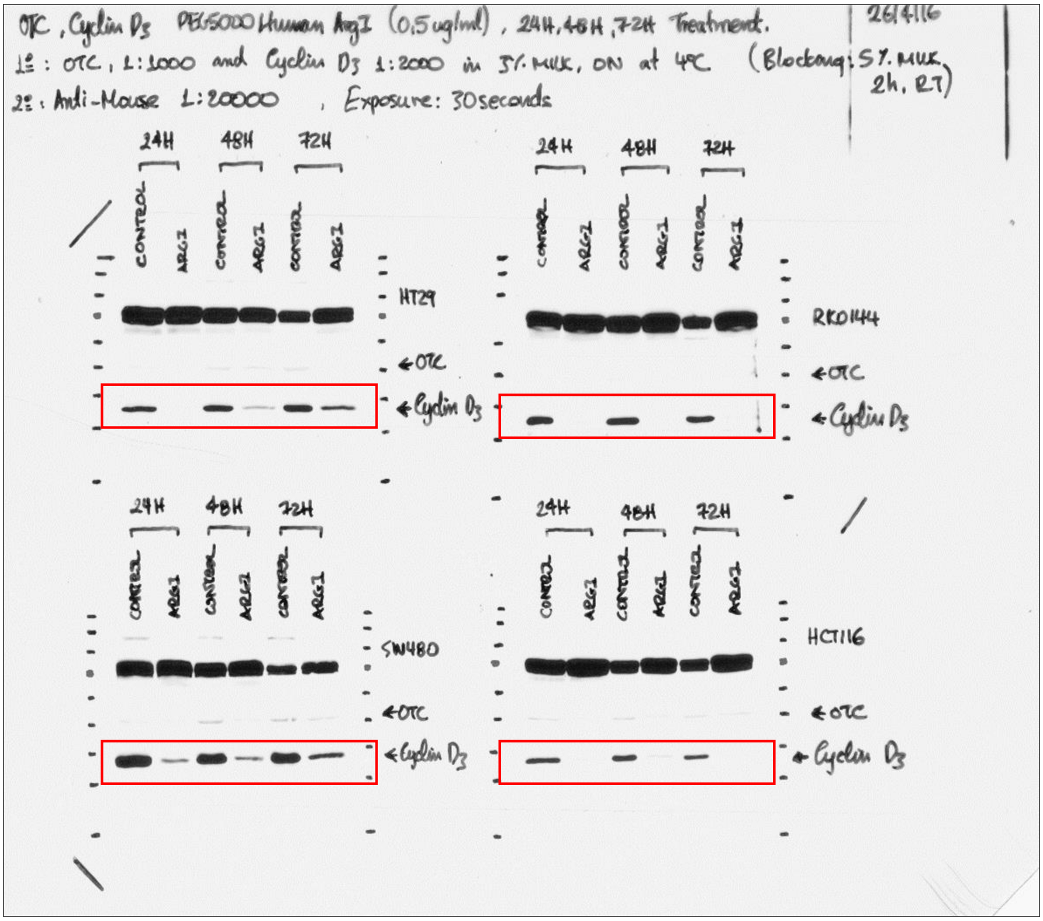

Figure S15

Western blots relative to Figure 4 –RKO xenografts

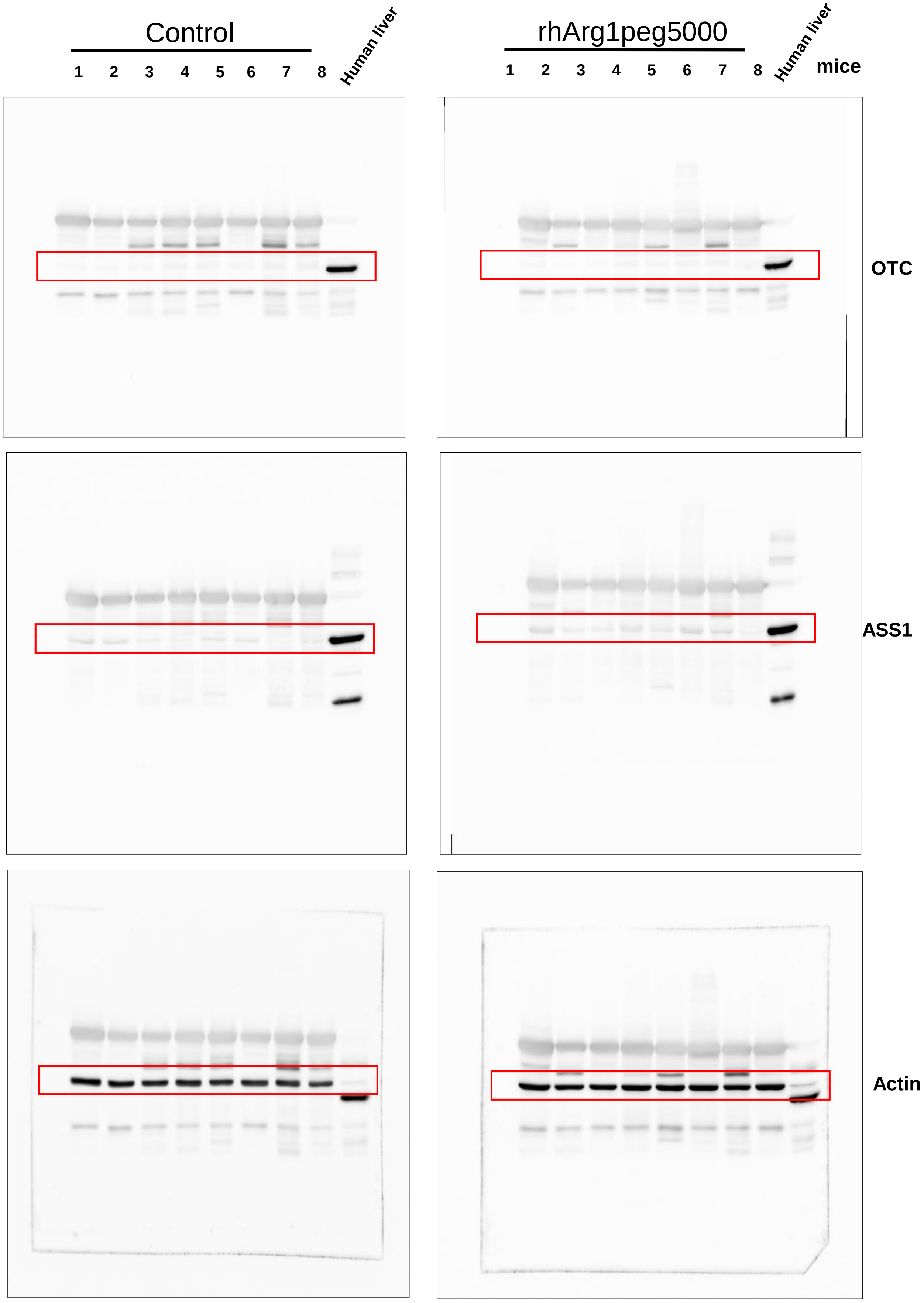

Figure S15

Western blots relative to Figure 4 –SW480 xenografts

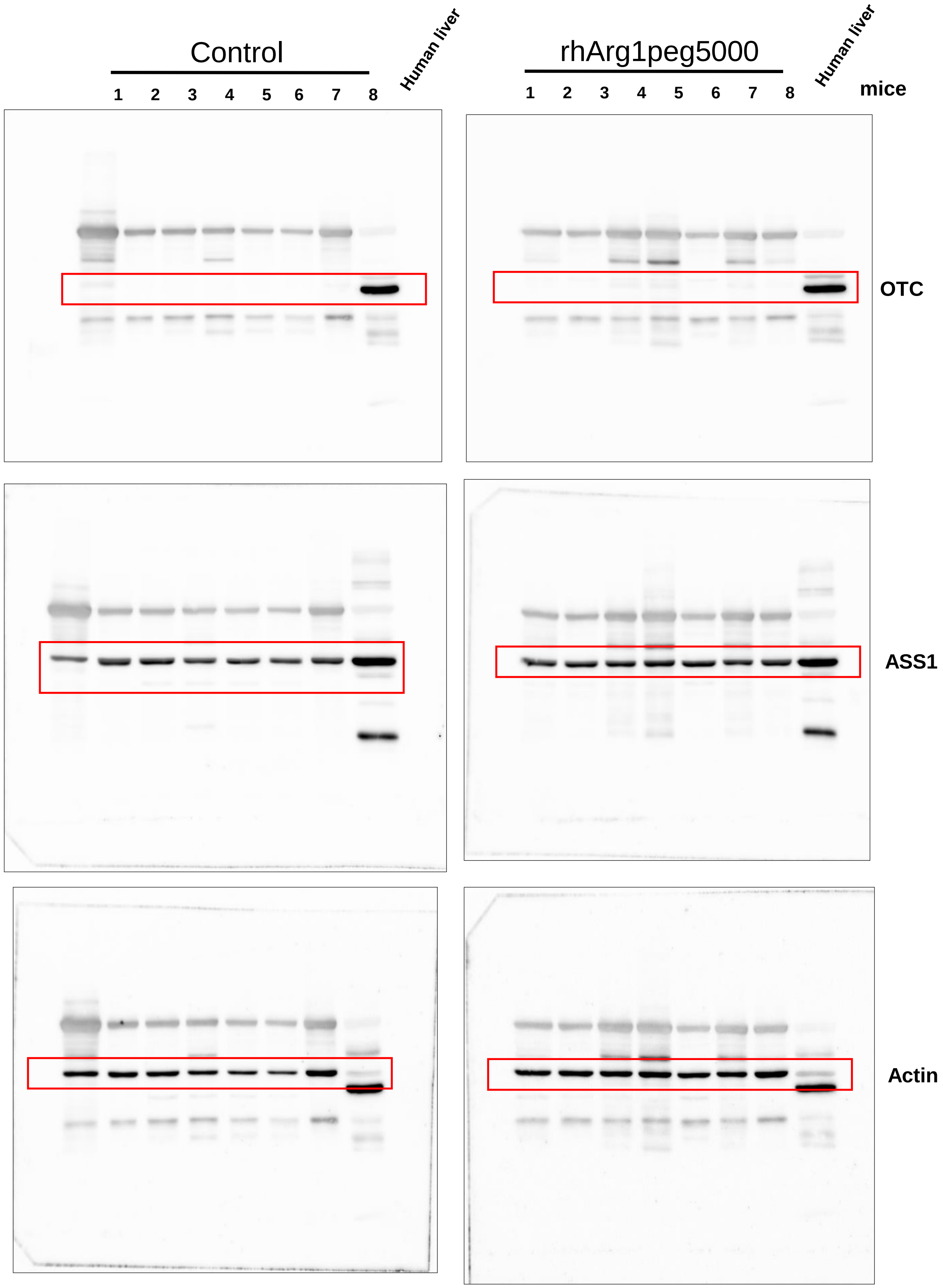

Figure S15

Western blots relative to Figure 5

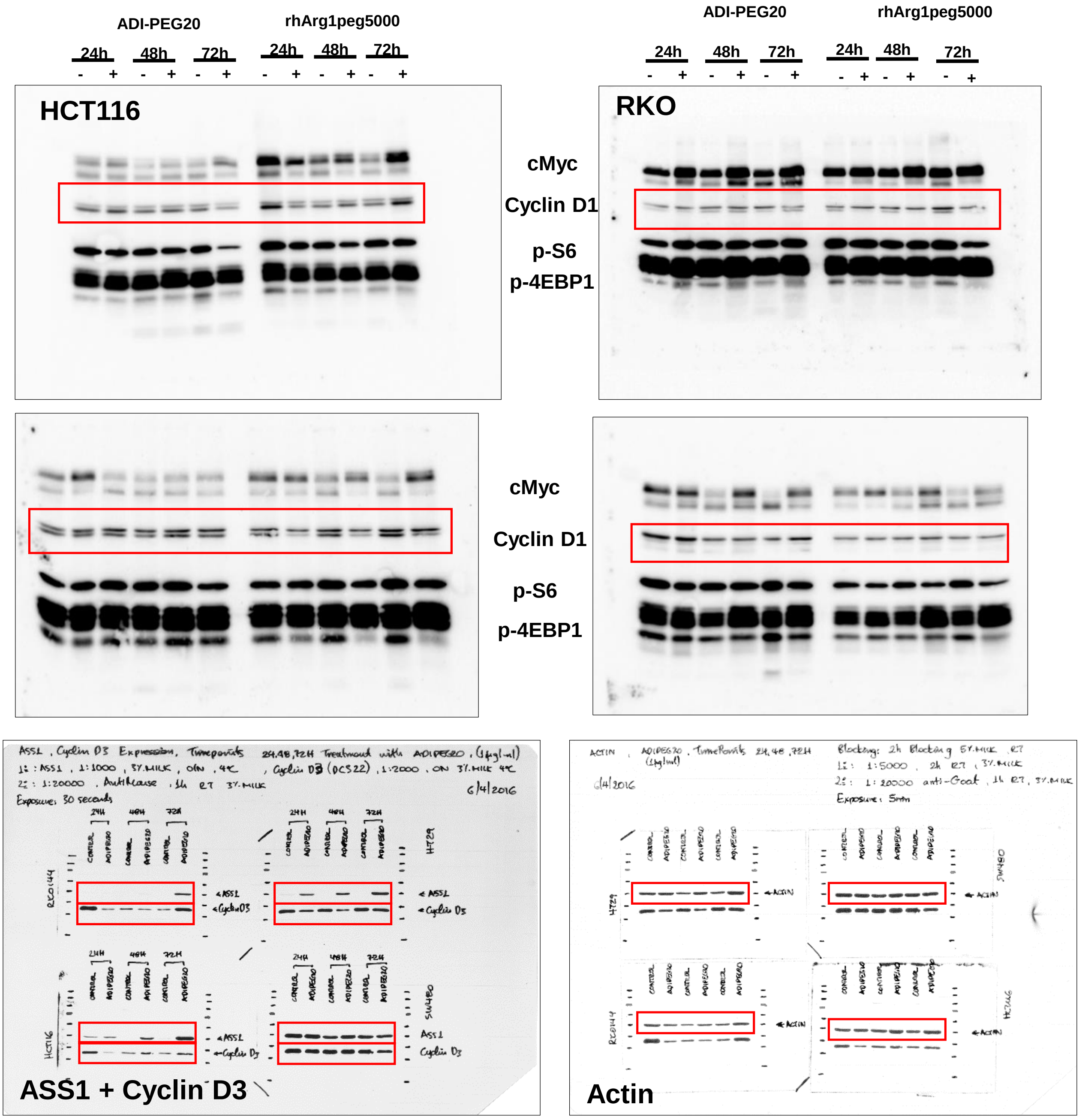

Figure S15

Western blots relative to Figure 6 –RKO xenografts

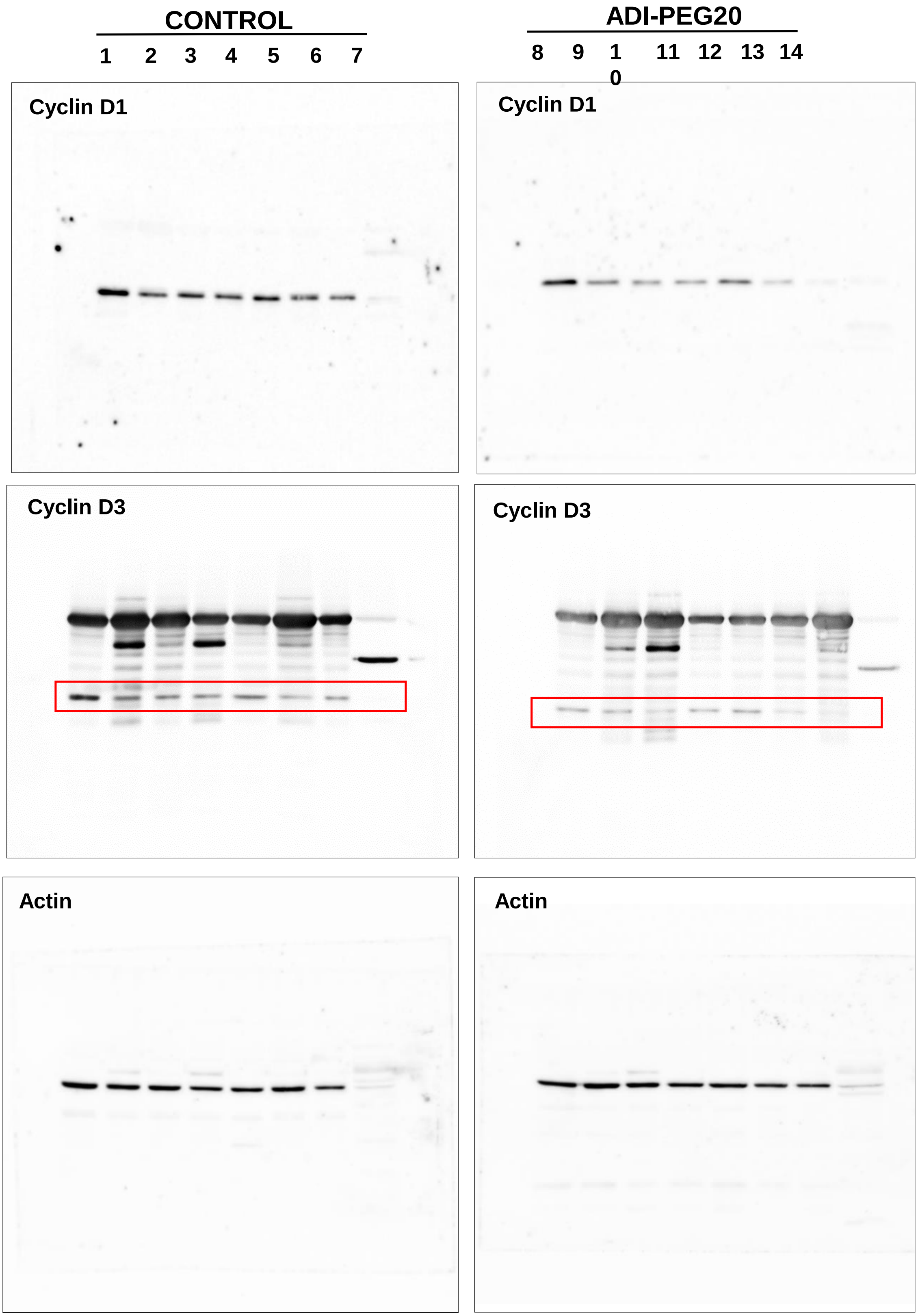

Figure S15

Western blots relative to Figure S2

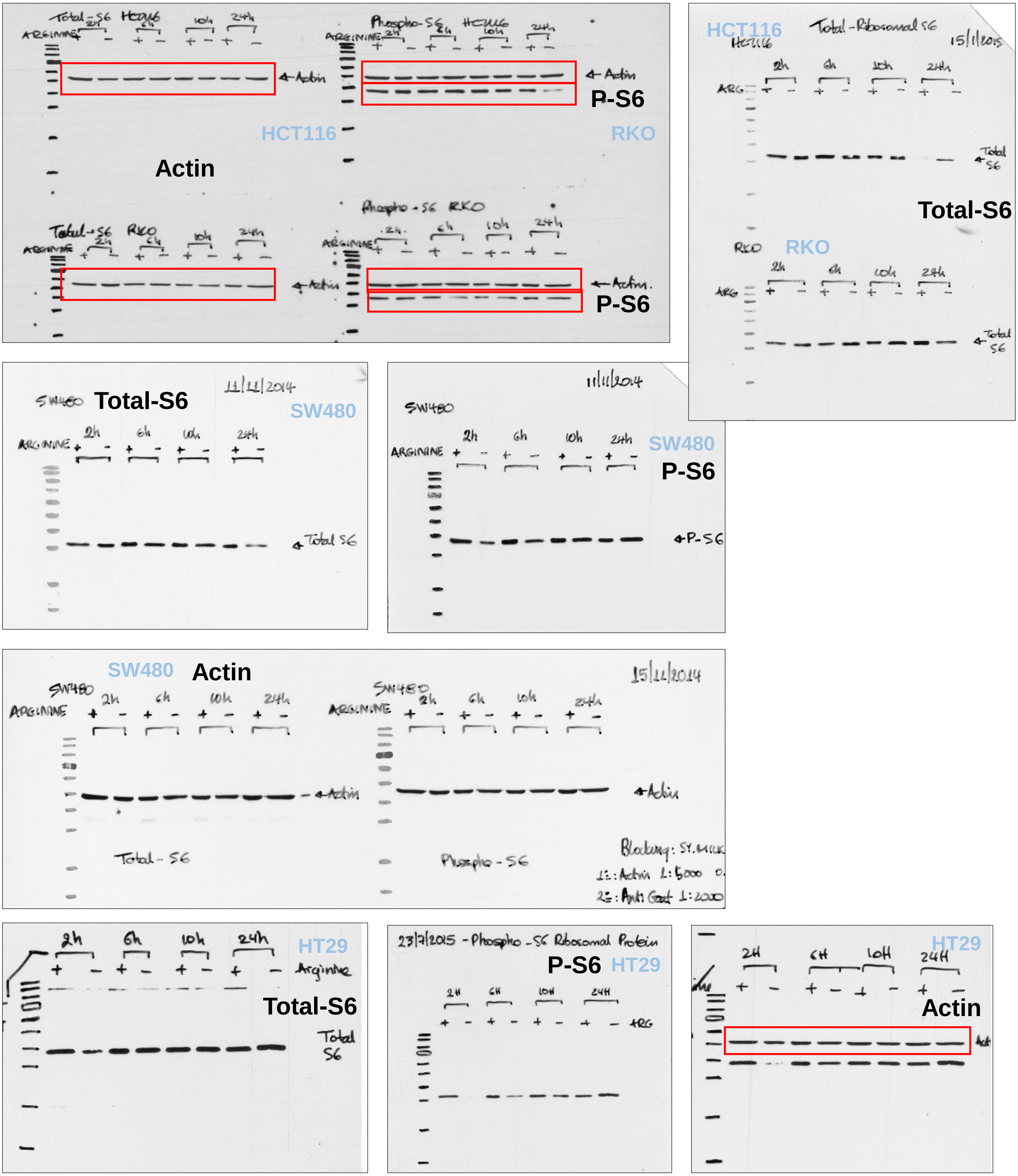

Figure S15

Western blots relative to Figure S2

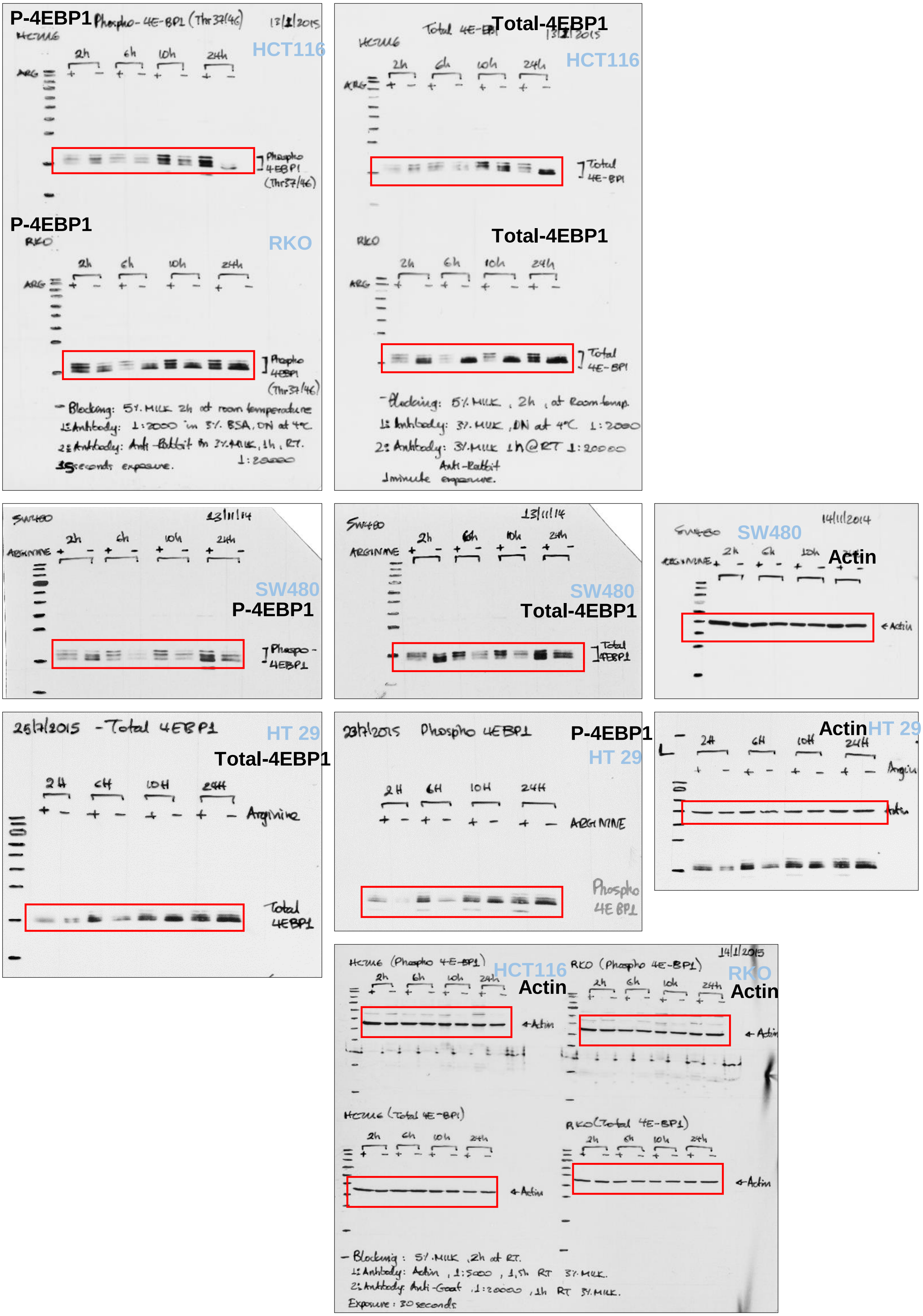

Figure S15 Western blots relative to Figure S6

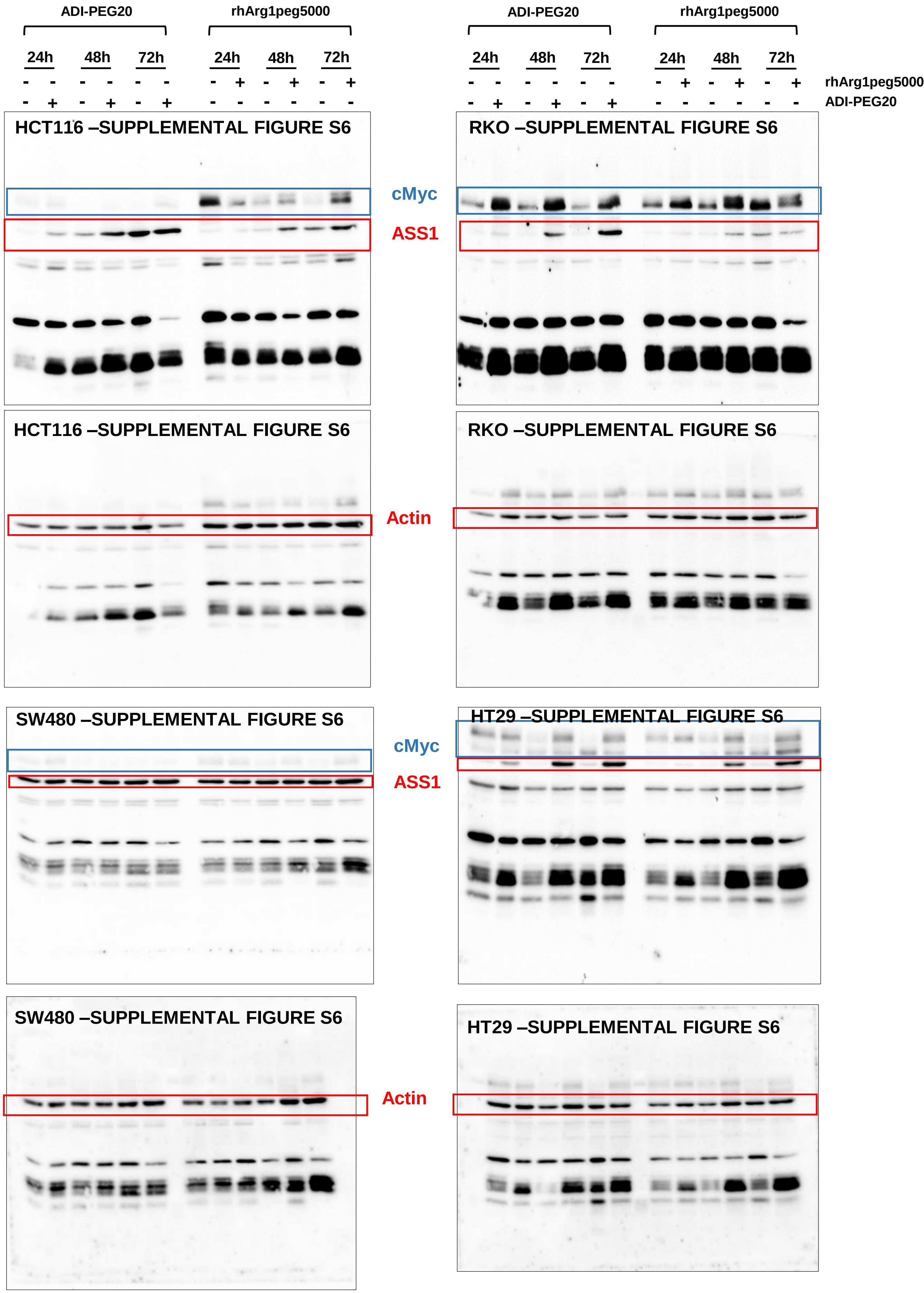

Figure S15

Western blots relative to Figure S7

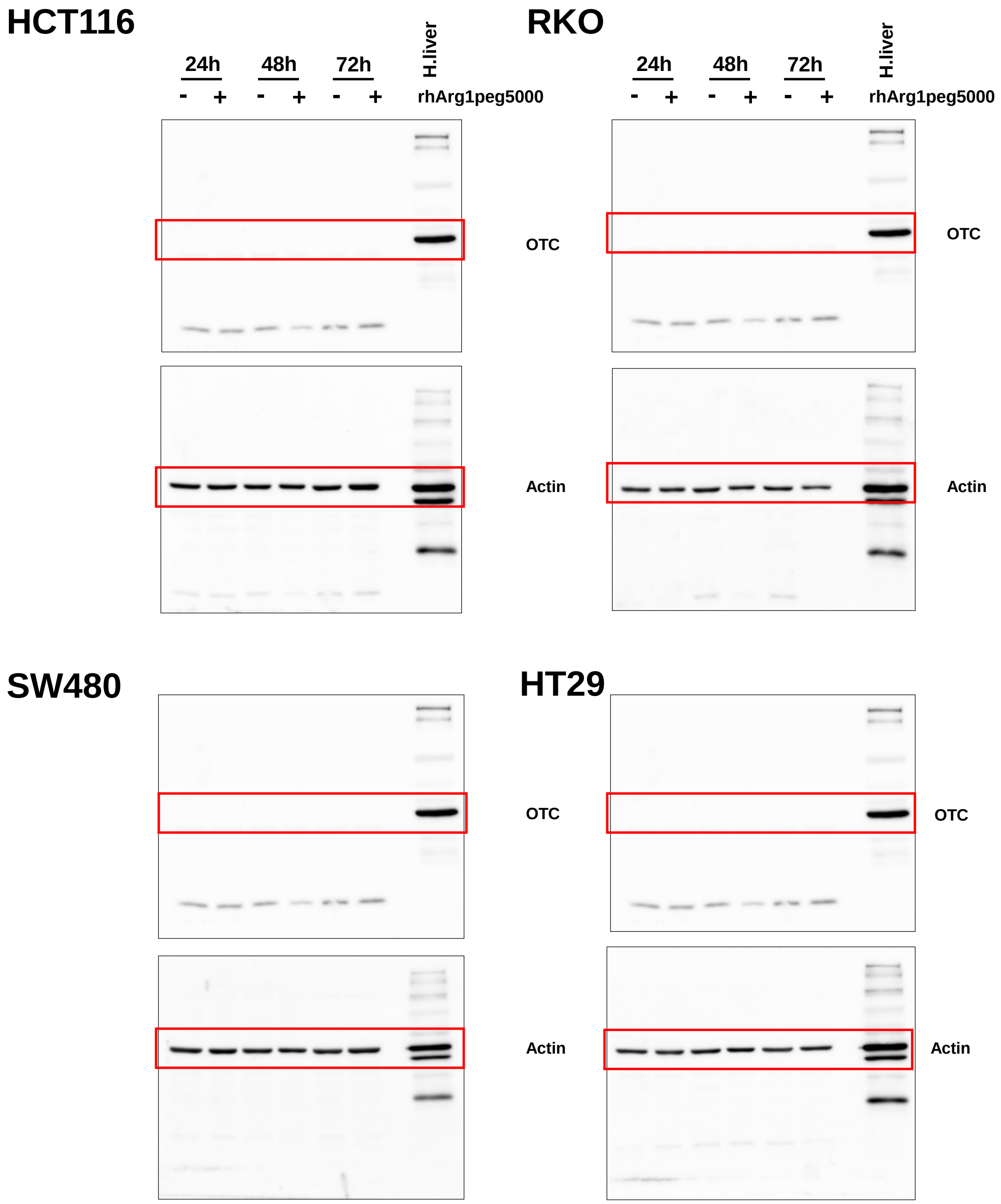

Figure S15

Western blots relative to Figure S8

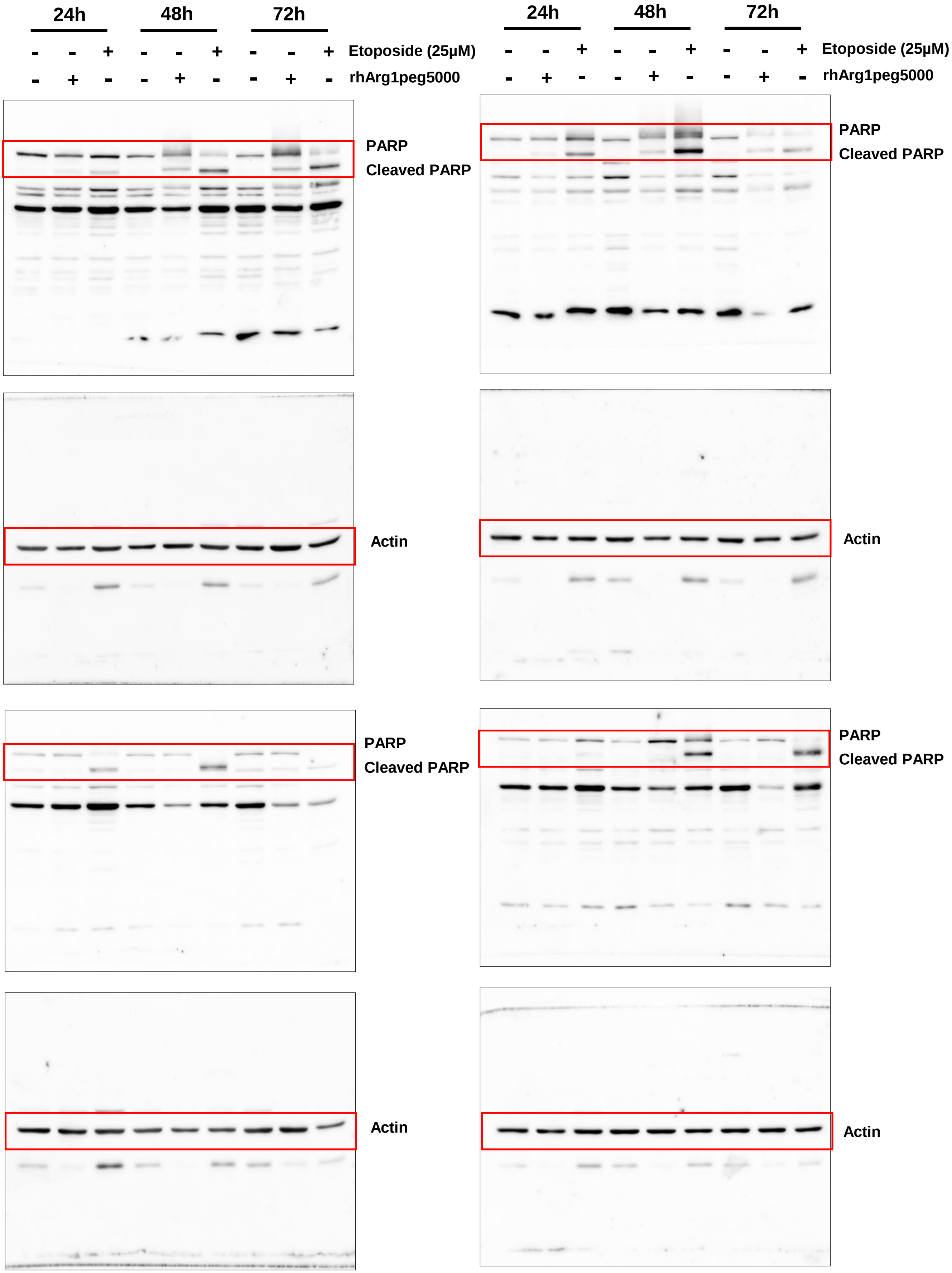

Figure S15

Western blots relative to Figure S9-HCT116

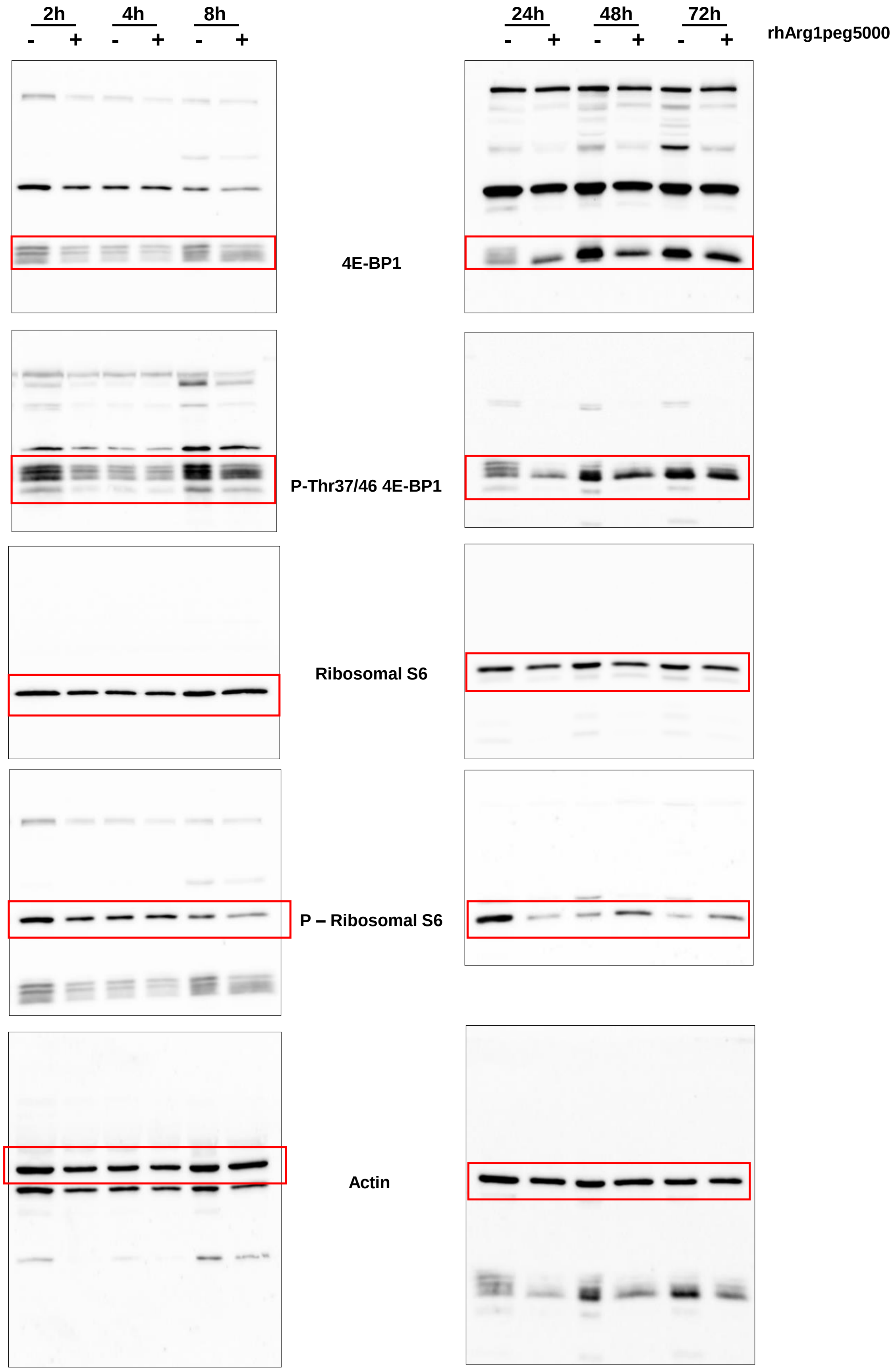

Figure S15

Western blots relative to Figure S9-RKO

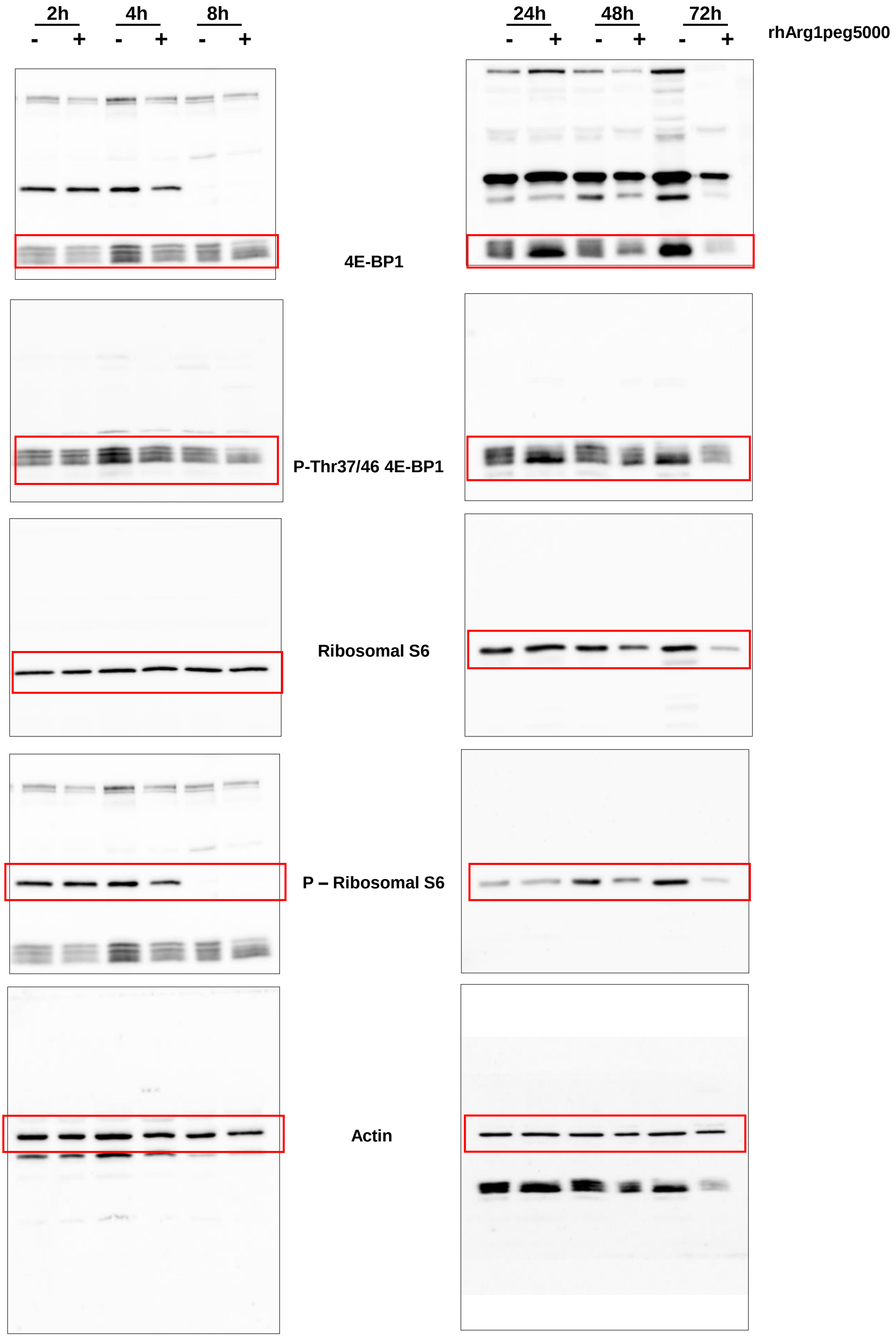

Figure S15

Western blots relative to Figure S9-SW480

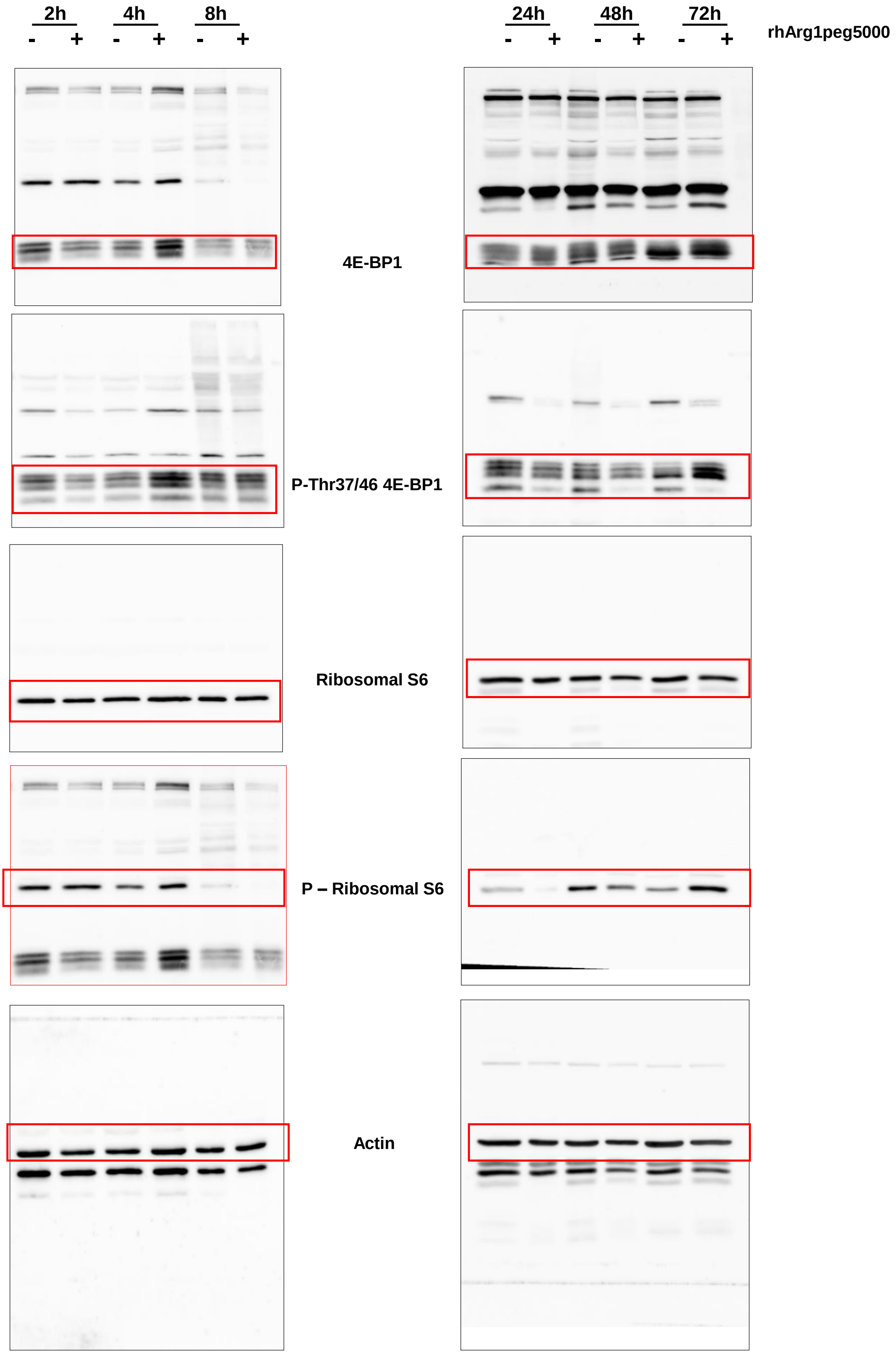

Figure S15

Western blots relative to Figure S9-HT29

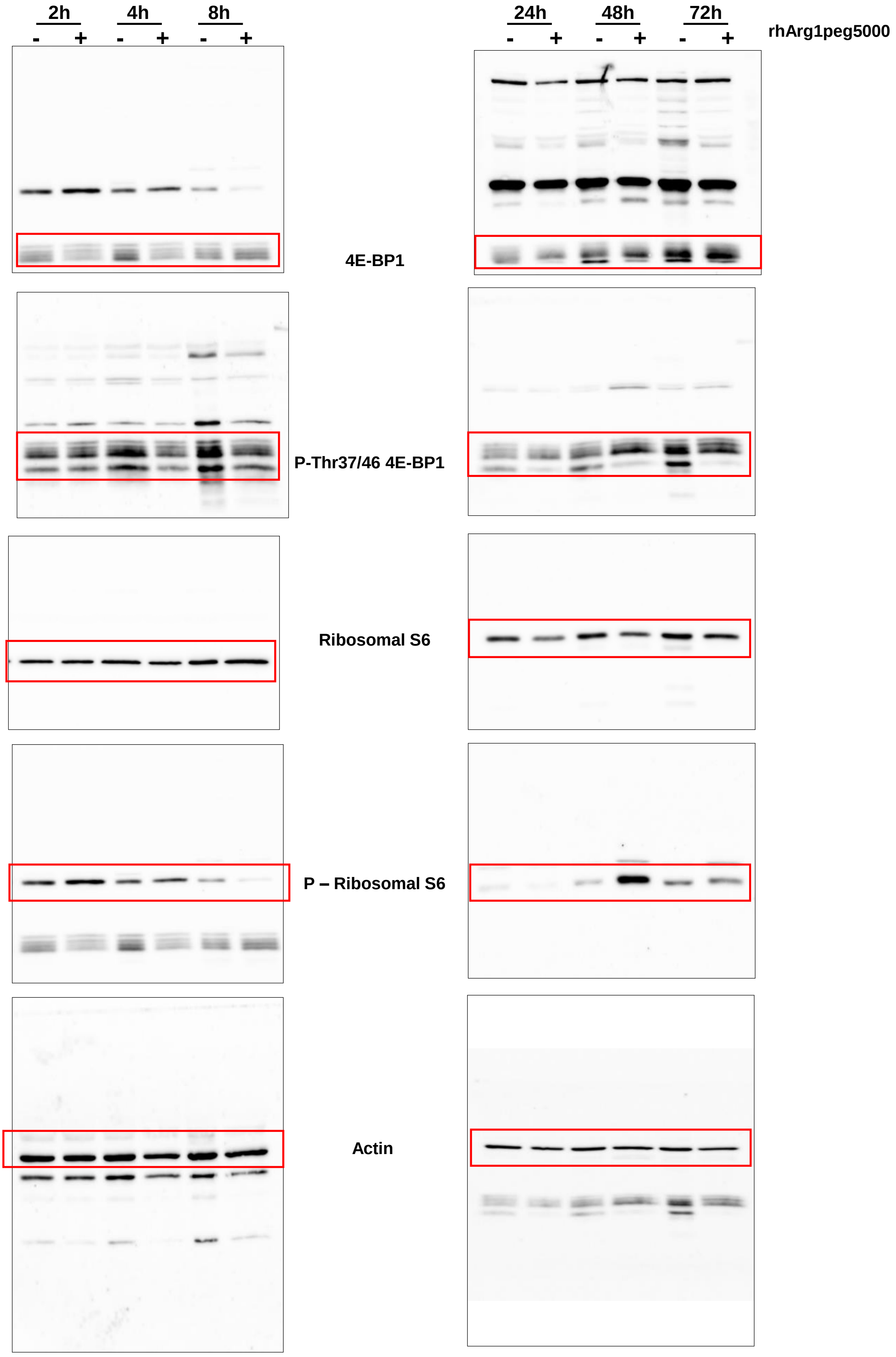

Figure S15

Western blots relative to Figure S11

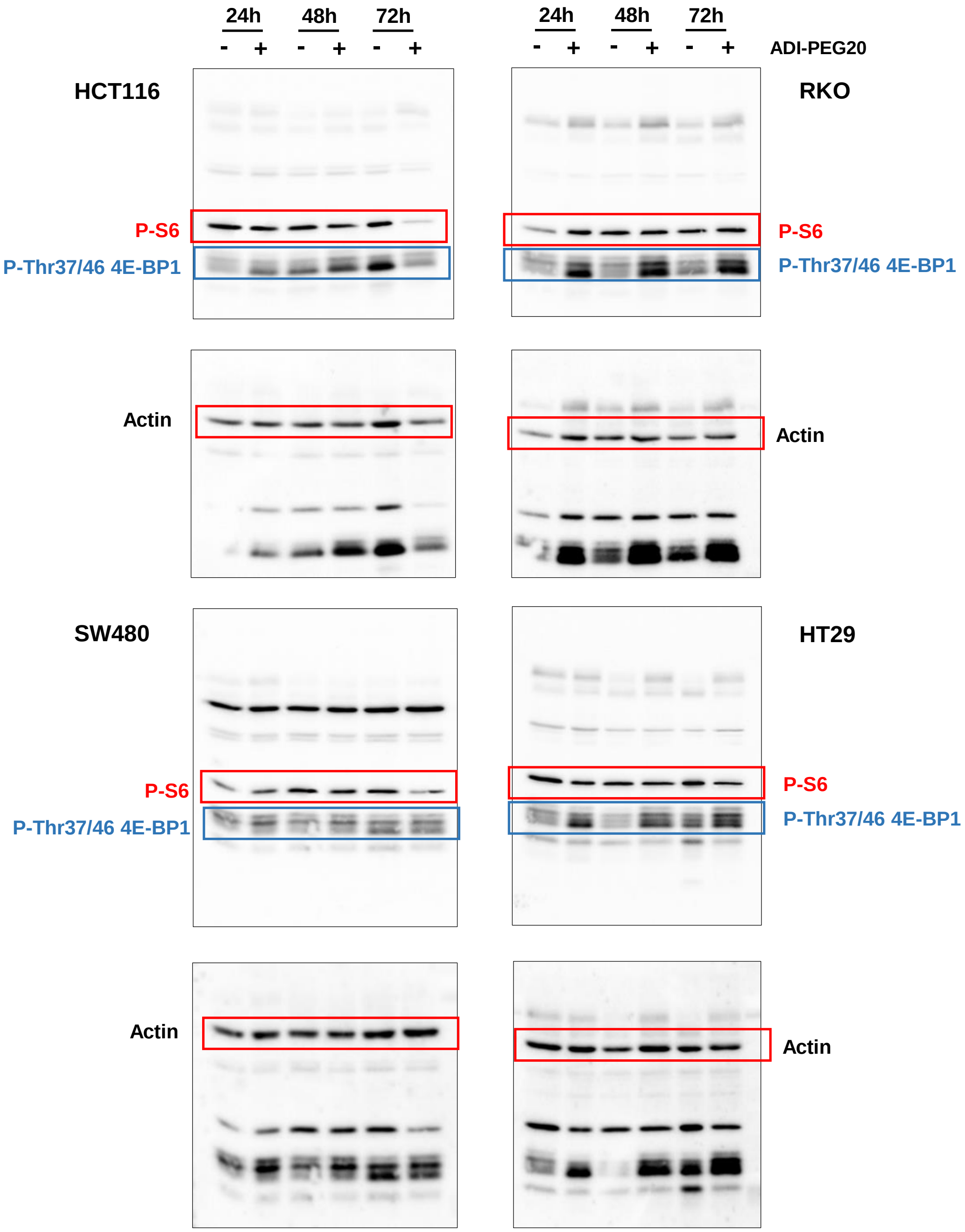

Characteristic	Number patients	Percentage (%)	Relationship with survival
		**Sex**	
Male	340	52.3	$\chi2$= 0.027, p=0.870
Female	310	47.7	
Age			
<70	305	46.9	$\chi2$=29.213, p<0.001
≥70	345	53.1	
		**Tumour site**	
Proximal colon	261	40.2	Proximal v distal, $\chi2$= 8.418, p=0.004
Distal colon	245	37.7	Distal v rectal, $\chi2$= 0.906, p=0.341
Rectum	144	22.2	Colon v rectum, $\chi2$=0.098, p=0.754
Tumour differentiation			
Well/moderate	600	92.3	$\chi2$=0.976, p=0.323
Poor	50	7.7	
		**Extra-mural venous invasion**	
Present	140	21.5	$\chi2$=100.946, p<0.001
Absent	510	78.5	
		**Mismatch repair protein status**	
Deficient	96	15.2	$\chi2$=2.848, p=0.091
Proficient	536	84.8	
		**pT stage**	
T1	30	4.6	T1 v T2, $\chi2$=0.382, p=0.536
T2	114	17.5	T2 v T3, $\chi2$=24.739, p<0.001
T3	411	63.2	T3 v T4, $\chi2$=30.159, p<0.001
T4	95	14.6	
		**pN stage**	
N0	364	56	N0 v N1, $\chi2$=54.071, p<0.001
N1	177	27.2	N1 v N2, $\chi2$=17.636, p<0.001
N2	109	16.8	
		**Dukes stage**	
A	120	18.5	A v B, $\chi2$=5.059, p=0.025
B	244	37.5	B v C, $\chi2$=65.510, p<0.001
C	286	44	

Supplemental Table 1. Clinico-pathological characteristics of patients in the TMA cohort.
